# Inactivation of folylpolyglutamate synthetase Met7 results in genome instability driven by an increased dUTP/dTTP ratio

**DOI:** 10.1093/nar/gkz1006

**Published:** 2019-10-24

**Authors:** Tobias T Schmidt, Sushma Sharma, Gloria X Reyes, Anna Kolodziejczak, Tina Wagner, Brian Luke, Anders Hofer, Andrei Chabes, Hans Hombauer

**Affiliations:** 1 DNA Repair Mechanisms and Cancer, German Cancer Research Center (DKFZ), Heidelberg D-69120, Germany; 2 Faculty of Bioscience, Heidelberg University, Heidelberg D-69120, Germany; 3 Department of Medical Biochemistry and Biophysics, Umeå University, Umeå SE-901 87 Sweden; 4 Institute of Developmental Biology and Neurobiology, Johannes Gutenberg Universität, 55128 Mainz, Germany; 5 Institute of Molecular Biology (IMB), 55128 Mainz, Germany; 6 Laboratory for Molecular Infection Medicine Sweden (MIMS), Umeå University, SE-901 87 Umeå, Sweden

## Abstract

The accumulation of mutations is frequently associated with alterations in gene function leading to the onset of diseases, including cancer. Aiming to find novel genes that contribute to the stability of the genome, we screened the *Saccharomyces cerevisiae* deletion collection for increased mutator phenotypes. Among the identified genes, we discovered *MET7*, which encodes folylpolyglutamate synthetase (FPGS), an enzyme that facilitates several folate-dependent reactions including the synthesis of purines, thymidylate (dTMP) and DNA methylation. Here, we found that Met7-deficient strains show elevated mutation rates, but also increased levels of endogenous DNA damage resulting in gross chromosomal rearrangements (GCRs). Quantification of deoxyribonucleotide (dNTP) pools in cell extracts from *met7Δ* mutant revealed reductions in dTTP and dGTP that cause a constitutively active DNA damage checkpoint. In addition, we found that the absence of Met7 leads to dUTP accumulation, at levels that allowed its detection in yeast extracts for the first time. Consequently, a high dUTP/dTTP ratio promotes uracil incorporation into DNA, followed by futile repair cycles that compromise both mitochondrial and nuclear DNA integrity. In summary, this work highlights the importance of folate polyglutamylation in the maintenance of nucleotide homeostasis and genome stability.

## INTRODUCTION

The one-carbon (1C) cycle is a central metabolic pathway that comprises several modification reactions of folates, which are used as 1C donors in a variety of biosynthetic processes. Folate cofactors are required for dTMP and purine biosynthesis (Supplementary Figure S1), glycine/serine homeostasis, homocysteine remethylation to methionine and the production of formyl-methionyl-tRNA that is necessary for the initiation of protein biosynthesis in bacteria, chloroplasts and mitochondria (1,2).

Due to the pivotal role of the 1C metabolism for cell proliferation and growth, drugs that target the 1C cycle (antifolates) have proved beneficial for treatment of cancer, autoimmune chronic diseases, as well as bacterial and parasite infections (3–7). Antifolates currently in use for cancer treatment inhibit dihydrofolate reductase (DHFR), that converts 7,8-dihydrofolate (DHF) into tetrahydrofolate (THF), the glycinamide ribonucleotide formyltransferase (GARFT) that uses 10-formyl-THF during the synthesis of purines, and thymidylate synthase (TS) that catalyzes the conversion of 2-deoxyuridine monophosphate (dUMP) into dTMP (8).

Antifolate treatment leads to a reduction in dTMP concentrations (with a consequent decrease in dTTP levels) and accumulation of dUMP (4,8). Studies in bacteria, yeast and human cells have shown that deprivation of thymine rapidly compromise cell viability, phenomenon known as *thymineless death* (TLD) (9–11). Despite that the underlying mechanism of TLD is not fully understood (12–14), substantial evidence indicates that a high dUTP/dTTP ratio drives uracil misincorporation into DNA causing genome instability (12,15). Since eukaryotic DNA replicative polymerases cannot distinguish between dTTP and dUTP (16), an increased dUTP/dTTP ratio promotes the incorporation of uracil (in place of thymine) during DNA synthesis. Misincorporated uracil triggers base excision repair (BER), that removes uracil from DNA; however, high uracil levels lead to reiterative uracil misincorporation/excision or ‘futile repair cycles’ resulting in frequent single and double strand breaks compromising genome integrity (17,18).

Under normal conditions, dUTP level is kept at extremely low concentrations, as dUTP is efficiently hydrolyzed into dUMP by the dUTP pyrophosphatase (Dut1) enzyme (Supplementary Figure S1). Accordingly, previous studies aiming to quantify dUTP levels in mammalian cells grown under normal conditions, either have failed (18–21), or have reported intracellular dUTP concentrations that differ several orders of magnitude between reports (11,22).

Complete loss of Dut1 activity in budding yeast causes lethality (23), whereas a *dut1* mutant (*dut1–1*) that retains ∼10% of the dUTPase activity shows increased uracil incorporation and genome instability (24). Up to now, *dut1–1* together with a hypomorphic thymidylate synthetase allele (*cdc21–1*) (25), are the only two reported genetic alterations in budding yeast associated with increased uracil incorporation.

Recently, we performed a genome-wide screen in *Saccharomyces cerevisiae* that identified a group of genes that strongly enhanced the mutator phenotype of strains expressing DNA polymerase active-site mutant alleles (26). In addition, we also identified 39 single gene deletions (not reported at that time) that confer a mutator phenotype in the presence of wild-type (WT) DNA polymerases. With one exception, all identified gene deletions affected well-characterized genes, most of them involved in distinct DNA repair pathways (27,28). The remaining identified hit was *MET7*, a gene that has not been previously associated with the suppression of mutations. *MET7* is a non-essential gene in *S. cerevisiae* that encodes for both the cytosolic and the mitochondrial folylpolyglutamate synthetase (FPGS) enzymes (29). In mammals, FPGS also exists as cytosolic and mitochondrial isoforms, but in contrast to Met7, its function is essential for survival of non-transformed proliferating cells (1,30). Met7/FPGS catalyzes the addition of up to eight glutamates (polyglutamyl tail) that are linked to the first glutamate in folate cofactors (Supplementary Figure S1). The polyglutamylation of folates is important for the 1C metabolism as it increases folate intracellular retention and enhances their affinity to folate-dependent enzymes (31). Furthermore, polyglutamylation is of clinical relevance, as human FPGS not only modifies folates but also antifolates that are frequently used for cancer treatment. Remarkably, a common mechanism of resistance to antifolate treatment in cancer cells occurs through the inactivation of human FPGS (5,8).

Previous studies in yeast reported that loss of *MET7* results in methionine auxotrophy (32), mitochondrial dysfunction (*petite* phenotype) (33), short telomeres (34–36), imbalanced dNTP pools and a defect in non-homologous end-joining (NHEJ) (36). However, the impact of Met7 and folate polyglutamylation on genome stability remains largely elusive in *S. cerevisiae*.

This study highlights the importance of Met7 to maintain nucleotide homeostasis, prevent uracil accumulation and consequently sustain the stability of both mitochondrial and nuclear DNA in budding yeast.

## MATERIALS AND METHODS

*Saccharomyces cerevisiae* strains used in this study (Supplementary Table S5) are derivatives of the S288c strains: RDKY3686 (*MATα ura3–52 leu2Δ1 trp1Δ63 hom3–10 his3Δ200 lys2–10A*) (37), RDKY5964 (a *MAT*a version of RDKY3686) (38), RDKY3615 (*MATa ura3–52 leu2Δ1 trp1Δ63 his3Δ200 lys2ΔBgl hom3–10 ade2Δ1 ade8 yel069c::URA3*) (39) or HHY6443 (RDKY5964 *iYEL072W::hph can1::hisG yel072w::CAN1/URA3 bar1::loxP.klLEU2.loxP*) (26). To further investigate the phenotype of the *dut1–1* mutation we performed some experiments (as indicated in Supplementary Figure S4) in the BY4741 (*MAT*a *his3Δ1 leu2Δ0 met15Δ0 ura3Δ0*)/BY4742 (*MATα his3Δ1 leu2Δ0 lys2Δ0 ura3Δ0)* background. Strains were cultivated in yeast extract-peptone-dextrose (YPD), yeast extract-peptone-glycerol (YPG) or synthetic media (SD) at 30°C according to standard protocols. Gene deletions and gene-tagging were performed using standard PCR-based recombination methods (40,41), followed by confirmation by PCR. Tags and junctions were confirmed by sequencing. Yeast strains expressing the *dut1–1* allele (*dut1-G82S*) (24) at the endogenous locus, were generated by pop-in/pop-out strategy with the integrative vector (pHHB1094). The presence of the *dut1-G82S* mutation, as well as the absence of additional unwanted mutations in this gene, was confirmed by sequencing (for details, see Supplementary Experimental Procedures).

### Identification of gene deletions causing mutator phenotypes in *S. cerevisiae*

We recently reported a genome-wide screen in which we identified factors that exacerbate the mutator phenotype of strains expressing active-site mutant DNA polymerases (26) or that result in a mutator phenotype in strains expressing wild-type DNA polymerases. In brief, HHY5298 (*MATa ura3–52 leu2Δ1 trp1Δ63 his3Δ200 lys2–10A cyh2-Q38K hom3–10.HIS3 pMFA1-klLEU2.hphNT1.lys2–10A MLH2.klURA3 POL1.natNT2)* was crossed against the non-essential gene-deletion collection (*MATα his3Δ1 leu2Δ0 ura3Δ lys2Δ yfg::kanMX4*) using a RoToR robot (Singer Instruments). Strains containing gene deletions in the presence or absence of DNA polymerase active-site mutations were tested for mutator phenotype with two *in vivo* mutational reporters (*lys2–10A* frameshift reversion assay (37) and *CAN1* inactivation assay (42)). Gene deletions that have not been previously reported to cause a mutator phenotype were validated by generating these knockouts *de novo* in RDKY5964 and HHY6443 for further analysis.

### Determination of mutation rates and GCRs

Mutation rates using the *CAN1* inactivation assay were determined in strains derived from RDKY5964 (38) by fluctuation analysis as previously described (43,44). Similarly, GCR rates were measured by fluctuation analysis in strains either derived from RDKY3615 (for the standard GCR assay) (39) or HHY6443 (for the post-duplication GCR reporter) (26). Mutation and GCR rates were determined based on two biological isolates and at least 14 independent cultures. 95% confidence intervals were calculated for all fluctuation tests.

### Determination of NTP and dNTP pools

NTP and dNTPs were initially measured as described before (45). Briefly, logarithmically growing yeast cells were harvested by filtration at a density of 0.4 × 10^7^ to 0.5 × 10^7^ cells/ml, disintegrated with a ice-cold mixture of 12% (wt/vol) trichloroacetic acid (TCA) and 15 mM MgCl_2_, and extracted with an ice-cold freon-trioctylamine mixture [10 ml of freon (1,1,2-trichloro-1,2,2-trifluoroethane); Millipore Sweden AB (>99%) and 2.8 ml of trioctylamine; Sigma–Aldrich Sweden AB (98%)]. 500 μl of the aqueous phase was treated with or without recombinant human dUTPase (hDut1, ab173062, Abcam) at a concentration of 1 ng/μl at 37°C for 1 h and then analyzed by strong ion exchange (SAX) high-performance liquid chromatography (HPLC) before (for NTP quantification) and after boronate chromatography (Affigel 601, Bio-Rad) (for dNTP quantification). Using this SAX-HPLC protocol, we could separate all NTPs and dNTPs except dUTP, which was only partially resolved from dTTP (Supplementary Figure S2A). The dTTP peak could still be quantified accurately since it is much larger than dUTP.

To resolve all NTPs and dNTP including dUTP, we developed a HPLC procedure based on reverse phase (RP) chromatography with tetrabutylammonium bromide as ion pairing agent. In this procedure, we introduced an extra step where the cell-containing filters (see above) were washed two times (30 ml each) with an ice-cold aqueous solution containing 8 g/l NaCl and 27 g/l glucose before disintegrating the cells with the TCA-MgCl_2_ solution. After the freon-trioctylamine step, the extracts were purified with OASIS-WAX (46), mixed 1:1 with mobile phase and separated at 0.5 ml/min on a 2.1 × 50 mm ACE Excel 2 μm C18-PFP column from Advanced Chromatography Technologies (Aberdeen, UK) using a UV-2075 Plus detector (Jasco International Co. Ltd, Hachioji, Japan) set at 270 nm (STD response time). A gradient between three aqueous solutions was used: solution A, B and C. Solution A contained 7% (v/v) methanol and 23 g/l KH_2_PO_4_ (HPLC grade from VWR International, Radnor, PA, USA), and the final solution was pH-adjusted to pH 5.6 with KOH. Solution B contained only 7% (v/v) methanol and solution C contained 7% (v/v) methanol and 3.52 g/l tetrabutylammonium bromide (ion-pair chromatography grade from Merck Group, Darmstadt, Germany). The run started isocratically with 10% A, 70% B and 20% C (min 0–10), followed by a linear gradient up to 60% A, 20% B and 20% C (min 10–27), and finally an isocratic step with 60% A, 20% B and 20% C (min 27–35), before returning to the initial conditions. The column was equilibrated for at least 15 min between the runs. Using the RP-HPLC protocol, we could separate all NTPs as well as ADP, dCTP and dUTP (Supplementary Figure S2B). However, co-purifying metabolites occluded the analysis of dGTP, and to some extent dTTP (as well as dATP in the WT extracts), and because of that, we excluded these peaks from the analyses. The dUTP peak was completely free from interfering peaks, and dCTP was nearly free from interference (only a minor peak was close to dCTP in the *met7Δ* mutant). In Supplementary Table S2, we have given the NTP and dNTP pools from the SAX-HPLC protocol and the NTP, dCTP, dUTP and ADP pools from the RP-HPLC protocol, whereas Figure 2A, B and Figure 3C show the results obtained from the SAX protocol. The high ATP/ADP ratio in all samples (∼20) indicates that the energy status was good and that the cells were not disturbed by the extra washing steps during harvesting. The quantification was performed by comparing the peak heights to a nucleotide standard for both protocols except for the NTPs in the RP protocol where areas were used instead. The reason for this is that the column needed to be slightly overloaded with NTPs (and then area is a more accurate measurement) in order to measure the much smaller dNTP peaks accurately.

### Yeast cell lysates and immunoblotting

*Saccharomyces cerevisiae* whole-cell protein extracts were generated as described (47) and analyzed on SDS-PAGE followed by immunoblotting using anti-Rad53 (EL7.E1, Abcam), anti-Rnr3 (AS09574, Agrisera), anti-tubulin/anti-Rnr4 (YL1/2, Sigma), anti-c-Myc (9E10, Millipore), anti-Clb2 (sc-9071, Santa Cruz Biotechnology), anti-Pgk1 (22C5D8, Invitrogen) and anti-Sic1 (this study).

### DNA content analysis

Logarithmic *S. cerevisiae* cultures were processed as described in (47) and analyzed using BD FACS Canto II (BD Biosciences) and FlowJo (v10.1, Tree Star Inc).

### Determination of growth rates

Yeast cultures were diluted to an optical density at 595 nm (OD_595_) of 0.05 by transferring the appropriate volume of an overnight culture into fresh YPD. Cultures were grown with shaking at 30°C for 12 h and OD_595_ measurements were taken every 30 min. Doubling times were calculated based on measurements obtained from at least two independent isolates per genotype.

### Live-cell imaging of Ddc2-GFP foci

Exponentially growing cells were processed and imaged as described in (38) using a Leica SP5 confocal microscope (Leica) with an Argon laser, a 63x 1.4NA objective and a high resonance scanner @8 kHz frequency. Ten 0.4 μm z sections were acquired; image processing such as maximum intensity projections were performed using ImageJ. Three independent biological replicates per genotype were analyzed and Mann–Whitney rank sum test was used to compare Ddc2-GFP foci abundance in WT and *met7Δ* strains. Statistical analysis was performed using the SigmaPlot software.

### Determination of uracil incorporation into genomic DNA

Uracil accumulation assay was mainly done as described (48). Genomic DNA was isolated from logarithmically growing cells using Puregene Yeast/Bact. Kit B (Qiagen) and further incubated overnight at 37°C with 10 U uracil DNA glycosylase from *E. coli* (UDG) and 20 U human AP endonuclease 1 (Ape1) (New England Biolabs) in 1× NEBuffer 4 (50 mM potassium acetate, 20 mM Tris-acetate, 10 mM magnesium acetate, 1 mM DTT, pH 7.9). DNA was precipitated and loaded on a 0.8% agarose gel stained with GelRed (Biotium). Images were taken using the GelDoc system (Bio-Rad).

### Telomere length analysis by southern blot

Genomic DNA (5 μg) was digested with XhoI for 5 h at 37°C. The digested DNA was separated on a 0.8% agarose gel overnight at 50 V. DNA in the gel was denatured for 1 h (0.4 M NaOH, 0.6 M NaCl) and neutralized for 1 h (1 M Trizma Base, 1.5 M NaCl, pH 7.4). DNA was transferred to a nylon membrane (Hybond NX, GE Healthcare) via capillary transfer in 10X SSC overnight and cross-linked to the membrane with UV light (auto X-link, Stratalinker). The membrane was pre hybridized for 5 h at 55°C in hybridization solution (PerfectHyb™ Plus Hybridization Buffer, Sigma). A telomere-specific probe was generated by random primed radioactive labeling with dATP [α-^32^P] (DECAprime kit II; Thermo Scientific) of a double-stranded DNA fragment obtained by digestion of pBL423 (a kind gift from M.P. Longhese (pSP100)) with EcoRI followed by gel extraction. Hybridization was carried out overnight at 55°C. The membrane was washed twice in 2× SSC with 0.1% SDS for 5 min and twice in 0.5× SSC with 0.1% SDS for 20 min. All washing steps were performed at 55°C. The signal was detected via Typhoon FLA 9500 (GE Healthcare).

## RESULTS

### Inactivation of the folylpolyglutamate synthetase Met7 results in mutator phenotype and GCRs

In a previous study, we identified a group of genes in *S. cerevisiae* that prevent the accumulation of mutations in strains expressing low-fidelity DNA polymerase alleles (26). As part of that study we also identified 39 genes (not reported at that time) that when inactivated in a WT strain caused an increased rate of mutations according to the *lys2–10A* frameshift reversion reporter and/or the *CAN1* inactivation assay (Supplementary Table S1). Among the genes that prevented frameshift mutations, we identified known components of the mismatch repair (MMR) system (*MSH2, MSH6, MSH3, MLH1, MLH3, PMS1* and *EXO1*) that participate at different steps during the correction of insertions/deletions or base substitutions (49–51). Moreover, we found that inactivation of Elg1, a subunit of an alternative replication factor C (RFC) complex (52,53) caused a mild increase in frameshift mutations (Supplementary Table S1). Elg1 promotes the unloading of Proliferating Cell Nuclear Antigen (PCNA) from DNA (54) and contributes in different aspects to the stability of the genome, including the suppression of frameshift mutations (27).

In addition, we identified 31 genes (e.g. *CCS1, CSM2, MET7, MMS2*, *MPH1*, among others) that upon inactivation caused an increased rate of *CAN1* inactivation, without compromising the repair of frameshift mutations at the *lys2–10A* reporter. With the exception of *MET7*, all other identified genes have been previously associated with the suppression of mutations (27,28,55–61). To validate the potential role of Met7 in the suppression of mutations we generated *de novo met7Δ* strains and measured the *CAN1* inactivation rate by fluctuation analysis. Supporting our initial observation, loss of Met7 resulted in a 9-fold increase (relative to WT) in the *CAN1* inactivation rate (Table 1).

**Table 1.** Inactivation of Met7 causes genome instability. Quantification of mutation rates and GCRs in several mutant strains

Relevant genotype	Mutation rate Can^R^ (fold increase)^a^	Post-duplication GCR (fold increase)^b^
WT	7.2 [5.7–9.0] x 10^–8^ (1)	5.6 [3.7–8.3] x 10^–8^ (1)
*met7Δ*	6.4 [4.2–8.8] x 10^–7^ (9)	9.9 [7.4–13.7] x 10^–6^ (177)
*pGPD-DUT1*	5.1 [3.1–7.5] x 10^–8^ (1)	4.4 [2.5–8.3] x 10^–8^ (1)
*pGPD-DUT1 met7Δ*	2.9 [1.9–5.5] x 10^–7^ (4)	4.6 [3.6–7.4] x 10^–7^ (8)
*ung1Δ*	3.5 [5.7–9.0] x 10^–7^ (5)	2.8 [1.6–6.2] x 10^–8^ (0.5)
*ung1Δ met7Δ*	6.9 [3.2–13.1] x 10^–7^ (10)	2.6 [2.0–3.5] x 10^–6^ (46)
*rev3Δ*	1.7 [1.0–2.5] x 10^–8^ (0.2)	not determined
*rev3Δ met7Δ*	3.0 [2.5–3.7] x 10^–7^ (4)	not determined
*dut1–1*	1.3 [1.0–2.5] x 10^–7^ (2)	1.2 [0.4–1.6] x 10^–7^ (2)
*dcd1Δ*	5.2 [3.2–6.8] x 10^–8^ (1)	3.6 [2.0–8.4] x 10^–8^ (1)
*dut1–1 dcd1Δ*	1.3 [0.6–1.8] x 10^–7^ (2)	6.3 [4.5–7.7] x 10^–7^ (11)

^a^Median rates of inactivation of *CAN1* gene (Can^R^) measured in strains derived from RDKY5964 (*CAN1* gene is located at its endogenous locus). *CAN1* inactivation rates are indicated with 95% confidence interval in square brackets and fold increase relative to the wild-type in parentheses.

^b^Median rates of GCRs (Can^R^ 5-FOA^R^ progeny) measured in strains derived from HHY6443 containing the post-duplication GCR reporter. Rates are indicated with 95% confidence interval in square brackets and fold increase relative to the wild-type in parentheses.

As inactivation of the *CAN1* gene can occur due to mispaired bases, frameshifts but also as result of GCRs, we tested whether loss of Met7 may also cause chromosomal instability. For this analysis we used yeast strains that harbor in the non-essential left arm of chromosome V two counter-selectable genes (*URA3* and *CAN1*) that confer sensitivity to 5-fluoroorotic acid (5-FOA) and canavanine, respectively. By measuring the spontaneous appearance of 5-FOA- and canavanine-resistant colonies (5-FOA^R^/Can^R^) in multiple independent cultures, it is possible to calculate the spontaneous inactivation rate of both genes, which is mainly due to GCR events. Quantification of GCR rates was done using yeast strains either carrying the ‘standard’ GCR reporter (39) or the ‘post-duplication GCR reporter’ (62). In the latter GCR reporter, GCR events are driven by a 4.2 kb region (*HTX13 DSF1*, located centromeric to the *URA3* and *CAN1* genes), that shares high sequence homology to sequences present in chromosomes XIV, IV and X, mainly resulting in homologous recombination-mediated GCRs. GCR rates measured in the WT strain using the standard- and post-duplication-GCR assay, were consistent with previous reports (39,47,62). Remarkably, Met7 inactivation resulted in a 39-fold increase in GCRs using the standard GCR assay (5.1 × 10^–11^ and 2.0 × 10^–9^ Can^R^ 5-FOA^R^ mutations per cell per generation in the WT and *met7Δ* strains, respectively) and 177-fold increase in the post-duplication GCR assay compared to the WT strain (Table 1). Therefore, loss of Met7 not only causes a mutator phenotype but also results in an increased GCR rate.

### Inactivation of Met7 causes activation of the DNA damage response

**Figure 1.**
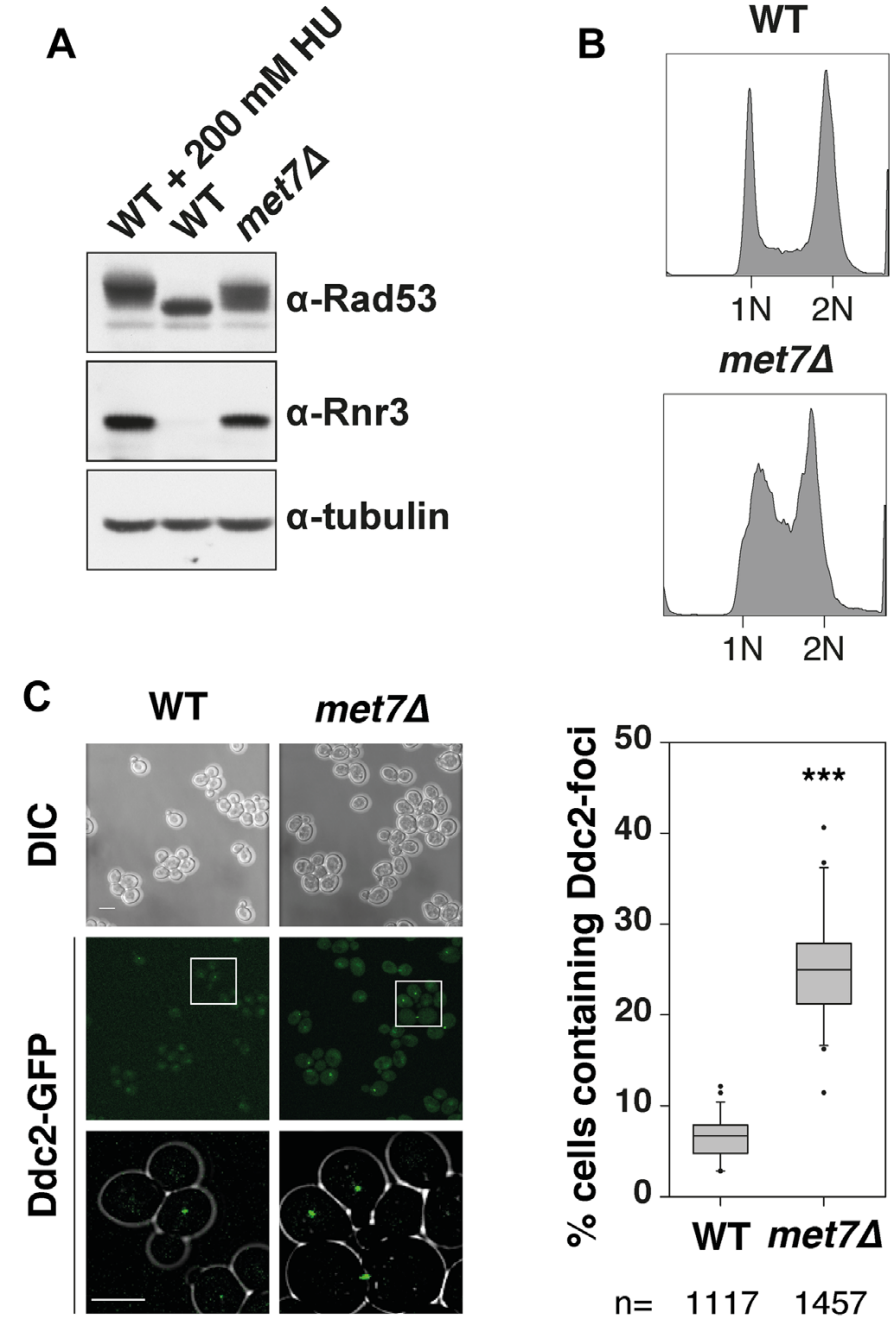
Inactivation of Met7 triggers activation of the DNA damage response. (**A**) Whole-cell lysates of WT and *met7Δ* strains were analyzed by western blotting with antibodies against Rad53 and Rnr3. WT cells incubated for 3 h in the presence of 200 mM HU were used as positive control for DNA damage/replication stress. Tubulin was used as loading control. (**B**) DNA content profile of logarithmically growing WT and *met7Δ* strains. (**C**) Quantification of Ddc2-GFP nuclear foci in WT and *met7Δ* strains. Representative images of differential interference contrast (DIC) and confocal fluorescent microscopy (Ddc2-GFP) are shown. Scale bar represents 5 μm. On the right, quantification of cells containing Ddc2-GFP foci in a box-plot with whiskers (indicating the 25^th^ and 75^th^ percentiles) and dots representing outliers and the line inside the box represent the median. (***) *P* < 0.001, statistical analysis using a Mann-Whitney rank sum test. ‘n’ indicates total number of cells imaged per genotype.

The slow growth phenotype of the *met7Δ* strain (33) and the increased genome instability phenotype, prompted us to investigate whether loss of Met7 triggers the activation of the DNA damage response. After analyzing cell lysates from logarithmically growing WT and *met7Δ* strains by western blotting, we found that loss of Met7 resulted in phosphorylation of the DNA damage checkpoint kinase Rad53 (phosphorylated forms of Rad53 give slower electrophoretic mobility bands), and up-regulation of the DNA damage-inducible ribonucleotide reductase (RNR) subunit Rnr3 that contributes to dNTP biosynthesis (Figure 1A). These findings, together with the accumulation of cells in S phase observed in the *met7Δ* mutant (Figure 1B), indicate that loss of Met7 results in constitutive S-phase checkpoint activation, potentially as a consequence of endogenous DNA damage and/or DNA replication stress.

We hypothesized that the increased GCR rate and the S-phase checkpoint activation in the *met7Δ* mutant might be triggered by an increased rate of endogenous DNA damage resulting in unrepaired double strand breaks (DSBs). To test for the presence of endogenous DSBs, we visualized in WT and *met7Δ* cells the DNA damage checkpoint protein Ddc2, which is recruited to DSB sites (63). Accordingly, the *met7Δ* mutant showed an increased number of Ddc2-GFP foci (5-fold over WT) (Figure 1C), which is indicative of endogenous DNA damage. In addition, we found that the *met7Δ* strain is hypersensitive to the double-strand break inducing agent phleomycin (shown later on in Figures 3A and 4D), potentially due to a DNA repair defect or the saturation of one or more DNA repair pathways.

As polyglutamylation of folates facilitates several biosynthetic reactions of the 1C cycle required for purine and dTMP biosynthesis, we asked whether Met7 protein expression increases during S phase and/or is potentially induced upon DNA damage, similar as described for ribonucleotide reductase subunits Rnr1–4 (64,65). To follow Met7 expression levels throughout the cell cycle, strains expressing Met7–3xMyc (C-terminal tagged at the endogenous locus) were arrested in G1 with α-factor. Synchronized cells obtained at different time points after release from G1-arrest were used for cell lysate preparation. Western blot analysis revealed that Met7 expression did not change as cells progressed through the cell cycle (Supplementary Figure S3A). Furthermore, we found that neither DNA replication stress (induced by 3 h exposure to 200 mM hydroxyurea (HU)), nor DNA damage (caused by 3 h treatment with 5 μg/ml phleomycin), resulted in changes in Met7 expression levels (Supplementary Figure S3B). Therefore, in contrast to Rnr subunits, Met7 expression remains unchanged throughout the cell cycle and is not affected by DNA damage or replication stress.

### Inactivation of Met7 causes a high dUTP/dTTP ratio resulting in uracil incorporation into genomic DNA

**Figure 2.**
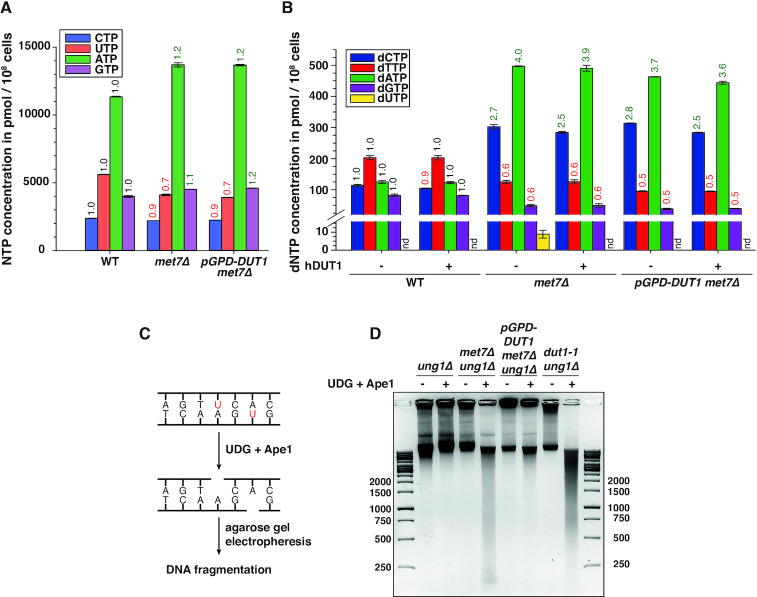
Inactivation of Met7 results in a high dUTP/dTTP ratio that causes increased uracil incorporation into DNA. HPLC quantification of NTP (**A**) and dNTP (**B**) concentrations in cell extracts of indicated strains. Numbers on top in green or red, indicate the fold increase or decrease, respectively, relative to WT levels. In (**B**), cell extracts were (+) or not (−) treated with recombinant human Dut1 prior quantification of dNTPs. ‘nd’ (or ‘not detectable’) indicates dUTP concentrations below our detection limit (≤3 pmol dUTP). (**C**) Schematic diagram illustrating approach used in (**D**) for the detection of incorporated uracil into genomic DNA. (**D**) Genomic DNAs isolated from the indicated strains were (or not) digested with UDG + Ape1 enzymes and subsequently visualized by agarose gel electrophoresis.

Previous work has shown that loss of Met7 results in a dNTP imbalance, mainly characterized by increased dCTP and dATP pools (36). Given the role of Met7 in promoting folate-dependent reactions that lead to the synthesis of purines and dTMP, we predicted that loss of Met7 might cause a reduction in purines and/or dTTP pools that could explain the activation of the DNA damage response (Figure 1A and B). As changes in dNTP pool homeostasis have been previously linked to mutator phenotypes in yeast (26,65,66) and mammalian cells (67,68), we analyzed whether loss of Met7 affects nucleoside triphosphate (NTP) and/or dNTP concentrations by HPLC. Measurement of NTP levels in the *met7Δ* mutant revealed a relative reduction of 10% and 30% in CTP and UTP levels, respectively, compared to WT (Figure 2A, Supplementary Tables S2A and S3A). In agreement with a previous report (36), we found that loss of Met7 resulted in increased dCTP and dATP pools (3- to 4-fold increase over WT). In addition, the *met7Δ* strain showed a 40% reduction in both dTTP and dGTP pools (Figure 2B and Supplementary Tables S2A and S3B), which was not previously reported. One possible explanation of this apparent disagreement could be due to the use of different yeast genetic backgrounds, or perhaps due to additional mutations that may arise given the genome instability phenotype associated with the *met7Δ* mutant strain. Interestingly, the dNTP imbalance observed in the *met7Δ* is reminiscent of dNTP pools measured in mammalian cells treated with antifolates that inhibit key enzymes of the folate cycle (e.g. DHFR and TS) (69–71), suggesting that dNTP pool alterations in the *met7Δ* mutant might be a consequence of folate depletion. As exposure of mammalian cells to antifolates not only results in a dNTP imbalance but also dUTP accumulation (4), we aimed to quantify dUTP levels in WT and *met7Δ* strains. Under normal conditions intracellular dUTP accumulation is prevented by the dUTPase Dut1, which dephosphorylates dUTP into dUMP (23). Thus, the intracellular dUTP concentration in proliferating WT cells is very low, making its quantification extremely difficult. Remarkably, in cell extracts from *met7Δ* strains we measured with the SAX-HPLC protocol 9 ± 2 pmol dUTP/10^8^ cells, whereas in WT cells dUTP levels were below our detection limit (≤3 pmol dUTP) (Figure 2B and Supplementary Table S2A). To further validate dUTP measurements, cell extracts were treated with recombinant hDut1 prior to dNTP quantification. Strikingly, dUTP was no longer detectable in hDut1-treated *met7Δ* samples, whereas no major additional changes were observed among other dNTPs (Figure 2B and Supplementary Table S2A). Likewise, *met7Δ* cells overexpressing the *DUT1* gene (*pGPD-DUT1*) under the control of a strong constitutive promoter showed undetectable dUTP levels and otherwise identical dNTP concentrations as measured in the *met7Δ* strain (Figure 2B and Supplementary Table S2A). To validate the results obtained by SAX-HPLC, we developed an alternative HPLC procedure based on reverse phase chromatography (RP-HPLC) with tetrabutylammonium bromide as an ion-pairing agent that allowed a more accurate quantification of dUTP pools, corresponding to ≤1 and 11 pmol dUTP/10^8^ cells in WT and *met7Δ* strains, respectively (Supplementary Table S2B). Otherwise, SAX-HPLC and RP-HPLC methods gave similar results for NTPs and dNTPs concentrations (Supplementary Table S2). The conclusion from these experiments is that loss of Met7 causes at least a 10-fold increase of intracellular dUTP levels.

Previous studies have shown that increased uracil incorporation observed upon folate depletion (e.g. induced by antifolates) causes genome instability (11,72). Therefore, we hypothesized that the underlying cause of the genome instability phenotype observed in the *met7Δ* strain might be related to a high dUTP/dTTP ratio that will lead to uracil misincorporation and futile repair cycles resulting in frequent DSBs. In order to test this hypothesis, we treated genomic DNA from WT and *met7Δ* strains with recombinant uracil DNA glycosylase (UDG) and apurinic/apyrimidinic endonuclease 1 (Ape1), and visualized its potential fragmentation pattern by agarose gel electrophoresis as previously described (48) (Figure 2C). Since uracil is efficiently removed from DNA by the uracil DNA glycosylase (Ung1), all strains used for this analysis were generated in strains lacking *UNG1*, which encodes for the nuclear and mitochondrial UDG isoforms (73,74). In addition, we included in our analysis the *dut1–1* mutant that has been previously shown to retain <10% dUTPase activity and to cause uracil incorporation into genomic DNA (24). Remarkably, while the DNA isolated from *ung1Δ* remained unchanged after UDG+Ape1 treatment, DNA isolated from the *met7Δ ung1Δ* mutant was vastly fragmented by UDG+Ape1 (with DNA fragments as low as 200 bases), indicative of massive uracil incorporation (Figure 2D). Similar results were obtained with DNA isolated from the *dut1–1* strain as previously reported (24). Importantly, the DNA fragmentation observed in the *met7Δ ung1Δ* strain was largely suppressed by *DUT1-*overexpression (Figure 2D), which is in line with the low dUTP levels in this strain (Figure 2B and Supplementary Table S2). Thus, inactivation of Met7 causes accumulation of intracellular dUTP levels resulting in increased uracil incorporation into DNA.

### Rev3 inactivation or Dut1 overexpression partially suppresses mutations in *met7Δ* strain

As uracil accumulates in the absence of Met7, we asked whether the increased uracil incorporation into DNA contributes to genome instability phenotype in the *met7Δ* mutant. The uracil DNA glycosylase Ung1 recognizes and removes the uracil moiety leaving behind an abasic site (75). Abasic sites are further processed by apurinic/apyrimidinic (AP) endonucleases, which generate a single strand break, which is subsequently repaired by short- or long-patch BER. If abasic sites are not processed by AP endonucleases, they cause stalled DNA replication forks during the next round of DNA replication, which leads to error-prone repair via the recruitment of translesion synthesis (TLS) DNA polymerases (75,76). To test the contribution of TLS polymerase Rev3 to the mutator phenotype observed in the absence of *MET7*, we determined the *CAN1* inactivation rate in the *met7Δ* strain in the presence and absence of *REV3*. Approximately, 50% of all *CAN1* inactivation events were *REV3-*dependent (Table 1). Moreover, *DUT1* overexpression in the *met7Δ* strain, which prevents dUTP accumulation and presumably to a large degree abasic site formation, also caused a 2-fold reduction in the *CAN1* mutation rate (Table 1). A similar reduction in the *CAN1* mutation rate was not seen when Ung1 was inactivated in the *met7Δ* strain. This last result suggests that Ung1-dependent uracil removal (and consequently the formation of abasic sites) is not the major source of *CAN1* inactivating mutations in the *met7Δ* strain. Moreover, no change in the *CAN1* inactivation rate was observed when Dut1 was overexpressed in the WT strain, suggesting that the lower mutation rate in the *pGPD-DUT1 met7Δ* mutant is linked to elevated dUTP pools that are normally not occurring in the WT strain.

To investigate which type of mutations occur in the absence of Met7, we analyzed the *CAN1* mutation spectrum in Met7-deficient strains. The mutational spectrum was dominated by base substitution mutations (77% of the *CAN1*-inactivation events), and among those we observed a 1.5-fold increase in the percentage of G-C to A-T mutations compared to the WT (Supplementary Table S4) (26). This increase in G-C to A-T transitions is likely a consequence of the reduced dGTP and dTTP, and elevated dATP levels observed in the *met7Δ* mutant (Figure 2B, Supplementary Tables S2A and S3B). This type of dNTP imbalance may result in C:A mispairs that in the next DNA replication cycle will lead to C to T transitions. No mutational hotspots were identified, and besides the slight increase in G-C to A-T transitions, the *met7Δ* mutation spectrum was not significantly different to the WT (Fisher exact test, *P* = 0.2275). Thus, *CAN1* inactivation events in the *met7Δ* mutant are likely driven by DNA replication errors due to imbalanced dNTP pools and by potential mutations arising from error-prone repair after uracil excision (75,77)

### Dut1 overexpression or Ung1 inactivation suppresses GCRs in the *met7Δ* mutant

Removal of uracil from genomic DNA can lead to the generation of single and double strand DNA breaks, that could explain the chromosomal instability phenotype observed in strains lacking *MET7* (Table 1). To test whether the increased GCR rate in *met7Δ* strains is a consequence of uracil accumulation/repair, we measured GCR rates (post-duplication GCR assay) in strains overexpressing *DUT1* or lacking *UNG1*, either in the WT or in the *met7Δ* background. Strikingly, the increased GCR rate in the *met7Δ* strains was suppressed to a large extent by Dut1-overexpression (from 177-fold down to 8-fold over WT levels), and to a lesser degree by Ung1 inactivation (from 177-fold down to 46-fold) (Table 1). On the other hand, *DUT1*-overexpression or Ung1 inactivation in a WT strain did not result in major changes in GCR rates. Thus, these results indicate that the elevated GCR rate in the *met7Δ* strain is triggered by uracil accumulation and can be suppressed either by reducing dUTP levels (Figure 2B and Supplementary Table S2) or by preventing the processing of uracil incorporated into genomic DNA (Figure 2D). Interestingly, these results are in agreement with the observation that increased dUTPase activity or inactivation of Ung1 protects yeast cells against toxic effects of antifolates or 5-fluorouracil exposure (48,72).

### Increased uracil incorporation in the *dut1-1* mutant has no major consequences on genome stability

Our results indicate that the genome instability phenotype of the *met7Δ* mutant is, at least in part, a consequence of a high dUTP/dTTP ratio that leads to uracil incorporation followed by futile repair cycles. We asked whether uracil incorporation is solely sufficient to drive elevated GCR rates, or if other additional factors may contribute to *met7Δ’s* genome instability phenotype. To answer this question, we measured mutation rates and GCR rates in the *dut1–1* strain, which like the *met7Δ* mutant showed increased uracil incorporation (Figure 2D). Interestingly, in contrast to the findings reported by (24), the *dut1–1* mutant despite its increased uracil incorporation phenotype, showed only a minor increase (2-fold) in both, the *CAN1* inactivation and GCR assays (Table 1).

To exclude the possibility of potential suppressor mutations that may have arisen during the generation of the *dut1–1* mutant, we re-introduced the *dut1–1* mutation in our S288c strain background, and also in an alternative yeast strain (BY4741/BY4742), and performed mating/sporulation and tetrad dissection analysis, followed by characterization of the obtained *dut1–1* mutants. Tetrad dissection analysis revealed that the *dut1–1* mutation did not cause an apparent growth defect in our S288c background, although it consistently resulted in colonies of reduced size in the BY-strain (Supplementary Figure S4A). These initial observations were complemented with growth rate analysis (Supplementary Figure S5), revealing in both yeast backgrounds slightly reduced growth rates in the *dut1–1* mutants, reflected on extended doubling times relative to the WTs (97 ± 0.7 versus 86 ± 0.6 min in the S288c background, and 95 ± 1.3 versus 86 ± 0.5 min in the BY-background). These results are in agreement with the growth defect previously described in the *dut1–1* mutant (24).

On the other hand, the qualitative mutator analysis in *dut1–1* mutant strains obtained after tetrad dissection, revealed no mutator phenotype in both tested genetic backgrounds (Supplementary Figure S4B and C), which supports our previous measurements (Table 1), and argues against the presence of unwanted mutations that may suppress the expected *dut1–1* genome instability phenotype (24). There are at least two possibilities that could explain this discrepancy: (i) The previously characterized *dut1–1* strain might contain an additional mutation that in combination with the *dut1–1* allele could result in a strong mutator phenotype. (ii) Alternatively, the *dut1–1* mutation may result in a mutator phenotype in specific genetic backgrounds, perhaps as result of differences in DNA replication fidelity and/or DNA repair efficiency.

**Figure 3.**
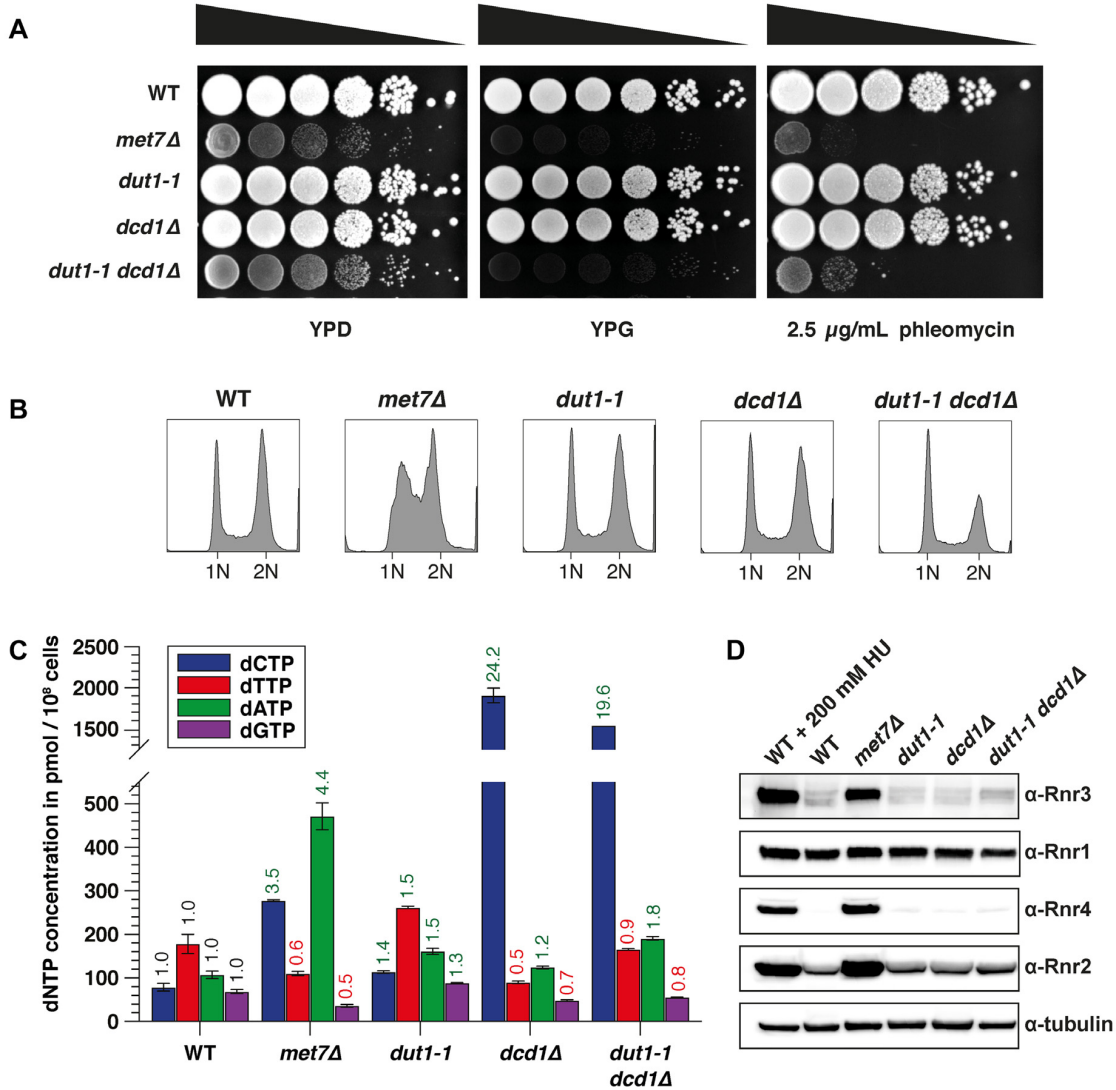
Increased genome instability in Met7-deficient strains is a consequence of uracil accumulation and limiting dNTP pools that may compromise DNA repair. (**A**) Yeast cultures of the indicated strains were serially diluted and spotted into YPD, YPG or YPD + 2.5 μg/ml phleomycin plates and grown at 30°C for 4–5 days. (**B**) DNA content analysis of logarithmically growing WT and mutant strains. (**C**) Quantification of dNTP pools in indicated strains. Data is based on the average of two biological replicates and shown as fold over WT. Numbers on top in green and red indicate the fold change in dNTPs relative to WT. (**D**) Whole-cell lysates of the indicated strains analyzed by western blotting with antibodies against Rnr1–4 subunits. Tubulin was used as loading control. WT cells treated for 3 h with 200 mM HU were used as positive control for an active DNA damage checkpoint.

In contrast to the *met7Δ* mutant, the *dut1–1* strain is neither a *petite* (indicated by the ability to use glycerol as carbon source), nor it is hypersensitive to DNA damage induced by phleomycin (Figure 3A). Furthermore, the *dut1–1* mutant, despite its slightly increased doubling time, showed a DNA content profile and intracellular dNTP pools, almost indistinguishable from the WT strain (Figure 3B-C and Supplementary Table S3). In agreement with these observations, cell lysates of the *dut1–1* strain analyzed by western blotting, showed WT-like levels of the DNA damage-inducible RNR subunits (Rnr3, Rnr2 and Rnr4), indicating that the DNA damage checkpoint is not activated (Figure 3D). Taken together, these results suggest that uracil incorporation alone might not be sufficient to cause increased mutagenesis or GCRs in budding yeast.

One difference between the *dut1–1* and the *met7Δ* strains is that the former one showed no reduction in dTTP levels. We speculate that the more severe dUTP/dTTP ratio in the *met7Δ* mutant might be responsible for the genome instability phenotype. In budding yeast, dTMP is synthesized by thymidylate synthase (Cdc21) (Supplementary Figure S1) that transfers a methyl group from 5,10-methylene-THF to the C5 position of dUMP to generate dTMP. Importantly, 60% of the dUMP pool is supplied by Dut1 through hydrolysis of dUTP, and 40% by deamination of deoxycytidine monophosphate (dCMP), a reaction catalyzed by the dCMP deaminase Dcd1 (24,78) (Supplementary Figure S1). For this reason, we asked whether a *dcd1Δ dut1–1* double mutant, if viable, might recapitulate the genome instability phenotype observed in the *met7Δ* strain. Interestingly, *dcd1Δ dut1–1* double mutant, similar to the *met7Δ* strain, grew poorly, showed phleomycin sensitivity and a *petite* phenotype, indicated by the inability to grow on media containing glycerol as carbon source (YPG plates) due to mitochondrial dysfunction (Figure 3A), but did not show an S phase delay or an activated DNA damage response (Figure 3B and D). Quantification of dNTP pools in the *dcd1Δ* single mutant revealed reduced dTTP and increased dCTP levels (Figure 3C and Supplementary Table S3B), which is in agreement with a previous report (78). However, in contrast to our expectations, the *dcd1Δ dut1–1* double mutant did not show lower but rather increased dTTP levels compared to the *dcd1Δ* single mutant (1.8-fold higher). In general, dNTPs in the *dcd1Δ dut1–1* double mutant approximate to the average of the *dcd1Δ* and the *dut1–1* single mutant dNTP concentrations. As the *dut1–1* single mutant shows slightly increased dNTPs (∼1.5-fold over WT), we propose that the *dut1–1* mutation causes an overall increase in dNTP levels in the *dcd1Δ* strain.

In addition, we found that the *dcd1Δ dut1–1* double mutant showed elevated GCR rates (11-fold over WT), but not as severe as in the *met7Δ* strain (177-fold increase over WT) (Table 1). These findings indicate that although both the *dut1–1* and the *met7Δ* mutant show increased uracil incorporation into genomic DNA (detectable in the absence of Ung1) (Figure 2D), *met7Δ’s* phenotype is by far more severe and complex than the phenotype caused by the *dut1–1* mutation (even in the absence of Dcd1), and therefore cannot be solely explained with a *thymineless death* model.

### Ung1 inactivation partially suppresses the *petite* phenotype in the *met7Δ* mutant

**Figure 4.**
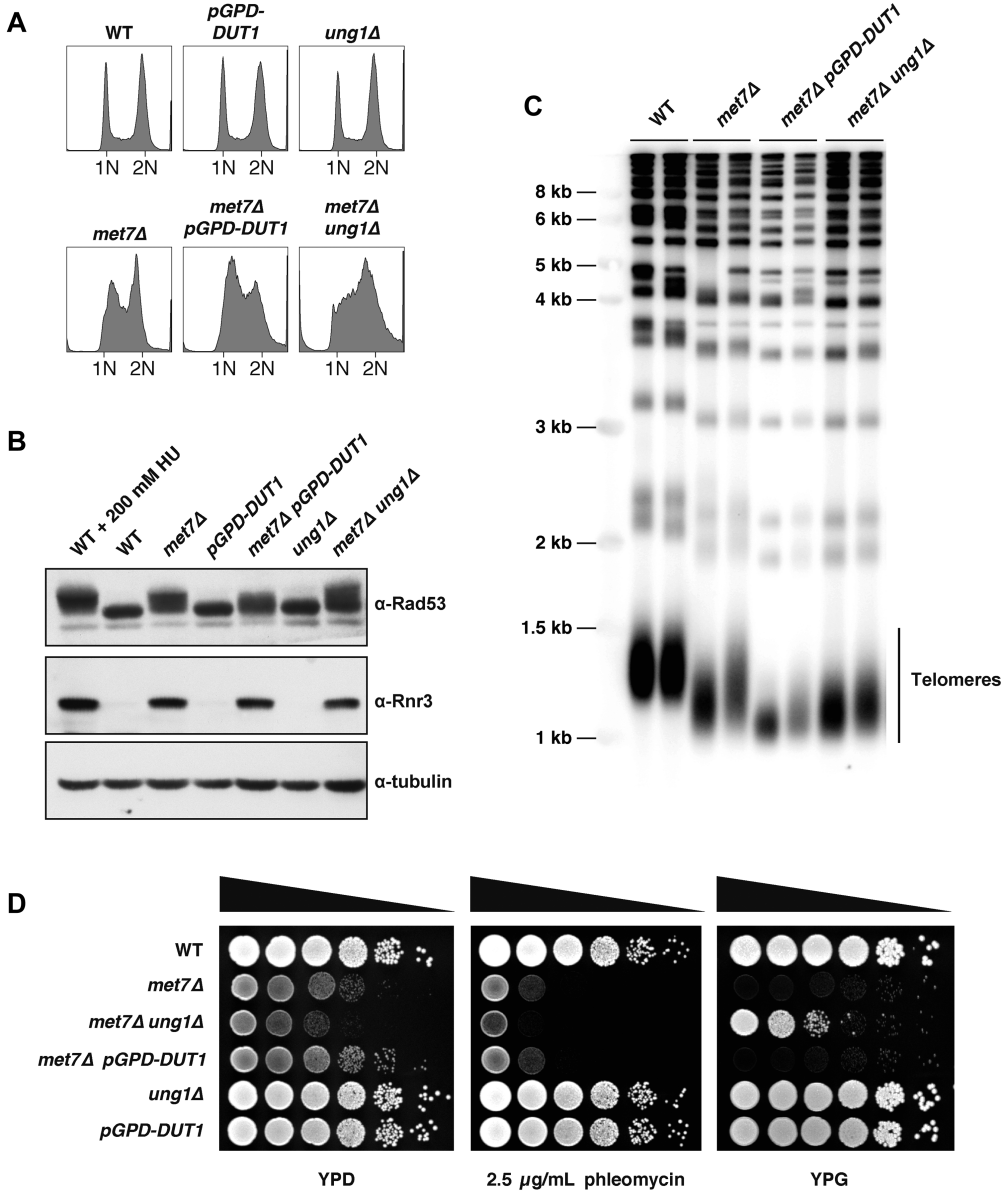
The *petite* phenotype in *met7Δ* mutant is partially suppressed by Ung1 inactivation. (**A**) DNA content profiles of indicated logarithmically growing yeast strains. (**B**) Whole-cell lysates of the indicated yeast strains were analyzed by western blotting with antibodies against Rad53, Rnr3 and tubulin (loading control). (**C**) Telomere-length analysis by southern blot of indicated yeast strains. (**D**) Cultures of the indicated strains were serially diluted and spotted into YPD, YPD + 2.5 μg/ml phleomycin or YPG plates and were grown at 30°C for 4–5 days.

Our results demonstrated that Dut1-overexpression or Ung1 inactivation largely suppressed the elevated GCR rates observed in the *met7Δ* strain (Table 1). Therefore, we asked whether other aspects of *met7Δ* phenotype (constitutive S-phase checkpoint activation, increased phleomycin sensitivity, short telomeres and *petite* phenotype) might be similarly suppressed by Dut1-overexpression and/or Ung1 inactivation. To answer this question, we first analyzed the DNA content profiles and the DNA damage response in the *met7Δ* strain under conditions that prevent uracil accumulation (*pGPD-DUT1*) or uracil removal (*ung1Δ*). Interestingly, we found that despite the fact that both mutations largely suppressed GCRs in the *met7Δ* mutant, neither one prevented S-phase checkpoint activation (Figure 4A and B). Similarly, short telomere and phleomycin sensitivity phenotypes of the *met7Δ* mutant were unchanged upon Dut1-overexpression or *UNG1* deletion (Figure 4C and D, respectively). Interestingly, the *met7Δ ung1Δ* double mutant (obtained after mating and sporulation), but not the *met7Δ pGPD-DUT1* strain, grew in YPG medium (Figure 4D), suggesting that *met7Δ’s* mitochondrial dysfunction might be in part a consequence of uracil misincorporation into mitochondrial DNA (mtDNA) followed by futile repair attempts mediated by Ung1. These results also suggest that Dut1 hydrolyzes mainly nuclear/cytoplasmic dUTP pools and might either have no access to mitochondrial dUTP, or might not be active throughout the cell cycle when mtDNA is replicated. As Dut1 prevents dUTP accumulation and at the same time provides dUMP for dTMP synthesis, the lack of mitochondrial dUTPase activity in budding yeast may explain why mtDNA is more sensitive to dTMP starvation than genomic DNA (10). This conclusion might not hold true for higher eukaryotes, as mammalian cells possess two different Dut1 isoforms, one constitutively expressed localized in the mitochondria, and a cell-cycle regulated isoform present in the nucleus (79).

## DISCUSSION

Here we report that loss of Met7, the folylpolyglutamate synthetase in budding yeast, results in severe genome instability, compromising both mitochondrial and nuclear DNA, leading to an increase in the mutation rate and GCRs. Met7-deficient cells accumulate dUTP, reaching levels that could be detected for the first time in yeast extracts (∼10 pmol dUTP/10^8^ cells, about 8-times lower than dGTP levels, the least abundant dNTP in WT cells). In contrast, dUTP concentration in WT yeast cell extracts is at least 10-times lower (≤1 pmol dUTP/10^8^ cells) and therefore is extremely difficult to quantify. This result is in agreement with several studies that could not determine intracellular dUTP levels in WT yeast or mammalian cells grown under normal conditions, either by HPLC, DNA polymerase-based assays or mass spectrometry (18–21).

Our experiments also demonstrated that genomic DNA isolated from the *met7Δ* mutant contained a substantial fraction of uracil indicated by the DNA fragmentation observed upon digestion with recombinant UDG+Ape1.

In general, the presence of uracil in DNA can be explained by two not mutually exclusive mechanisms: an elevated dUTP/dTTP ratio that favors uracil misincorporation during DNA replication, or the spontaneous/enzymatic deamination of cytosine bases in DNA (77,80). The observation that the *met7Δ* mutant showed increased accumulation of intracellular dUTP and reduced dTTP levels, and the fact that Dut1-overexpression largely suppressed dUTP accumulation and the presence of uracil in genomic DNA, strongly indicates that uracil detected in the *met7Δ* strain is mainly the result of dUTP misincorporation and not due to cytosine deamination events. Moreover, the elevated GCR rates in the *met7Δ* strain are linked to the removal of misincorporated uracil from genomic DNA. This is supported by the observation that the increased GCR rates can be suppressed by preventing uracil accumulation or its excision from DNA.

Given that the *met7Δ* and the *dut1–1* mutants both have increased uracil incorporation in DNA but only the former one showed constitutive activation of the DNA damage response, it is likely that the reduced dTTP and dGTP concentrations found in the *met7Δ* mutant result in the activation of the DNA damage response, rather than uracil incorporation (or its excision). This hypothesis is further supported by the observation that although Dut1 overexpression in *met7Δ* prevents dUTP accumulation and GCRs, it neither suppresses the S phase delay nor the DNA damage checkpoint activation. The fact that the *dut1–1* strain, despite its massive uracil incorporation, lacks a genome instability phenotype, indicates that uracil incorporation in the *dut1–1* mutant is not sufficient to drive genome instability, at least in the two genetic backgrounds tested in this study.

Previous reports in budding yeast have indicated that Met7 is necessary for the maintenance of an intact mitochondrial genome (33); however, given the pleiotropic consequences caused by the loss of Met7, it remained unclear how Met7 contributes to mtDNA stability. As Met7 supports the production of formyl-methionyl-tRNA required for the initiation of protein biosynthesis in the mitochondria (1,2), one possible scenario is that mitochondrial genome instability might arise as a consequence of a defect in mitochondrial protein synthesis. However, our observations in the *met7Δ* mutant, including the increased uracil incorporation into DNA, together with the partial suppression of its *petite* phenotype upon inactivation of Ung1, strongly argues that mitochondrial dysfunction is rather caused by elevated levels of uracil incorporation into DNA. Yet, is the mitochondrial dysfunction a direct consequence of increased uracil incorporation into mtDNA or might be an indirect effect caused by an unstable nuclear genome? The previous characterization of a thymidylate synthase mutant (*cdc21–1*), that similar to the *met7Δ* mutation, prevents dTMP biosynthesis and shows increased frequency of *petite* formation (81) supports the first of these two hypotheses. The authors found that *cdc21–1* mutants accumulate uracil in both genomic and mitochondrial DNA, and that the appearance of *petites* was dependent on the mitochondrial but not the nuclear isoform of Ung1. Thus, based on this previous study and our findings, we propose that the mitochondrial dysfunction in *met7Δ* mutant is rather a direct consequence of Ung1-dependent uracil excision repair cycles compromising mtDNA integrity.

The reduced telomere length in *met7Δ* strain is unlikely to be the consequence of DNA damage triggered by the incorporation of uracil into DNA, as neither Dut1-overexpression nor Ung1 deletion suppress the short telomere phenotype. Instead, this phenotype is most likely associated with limiting dGTP concentrations in this mutant strain. Previous studies reported a positive correlation between intracellular dGTP levels and both telomere length and telomerase processivity *in vivo* (82,83). Thus, our findings showing reduced dGTP levels in the *met7Δ* mutant that correlate with its short telomere phenotype support the hypothesis that telomerase activity is positively regulated *in vivo* by dGTP levels.

In this study, we also found that the *met7Δ* mutant shows a higher percentage of cells containing Ddc2-foci and increased sensitivity to the double-strand break inducing agent phleomycin. Together, these observations indicate that the *met7Δ* mutant accumulates endogenous DNA damage that may overload the DNA repair machinery or potentially compromise its function in an unrelated manner. The increased sensitivity to phleomycin in the *met7Δ* mutant seems not to be related to uracil accumulation, as it is not suppressed by the Dut1-overexpression. Instead, we propose that phleomycin sensitivity and the DNA repair defect (36) in the absence of Met7 are caused by the inability to increase dTTP and dGTP levels to facilitate DNA repair (84).

**Figure 5.**
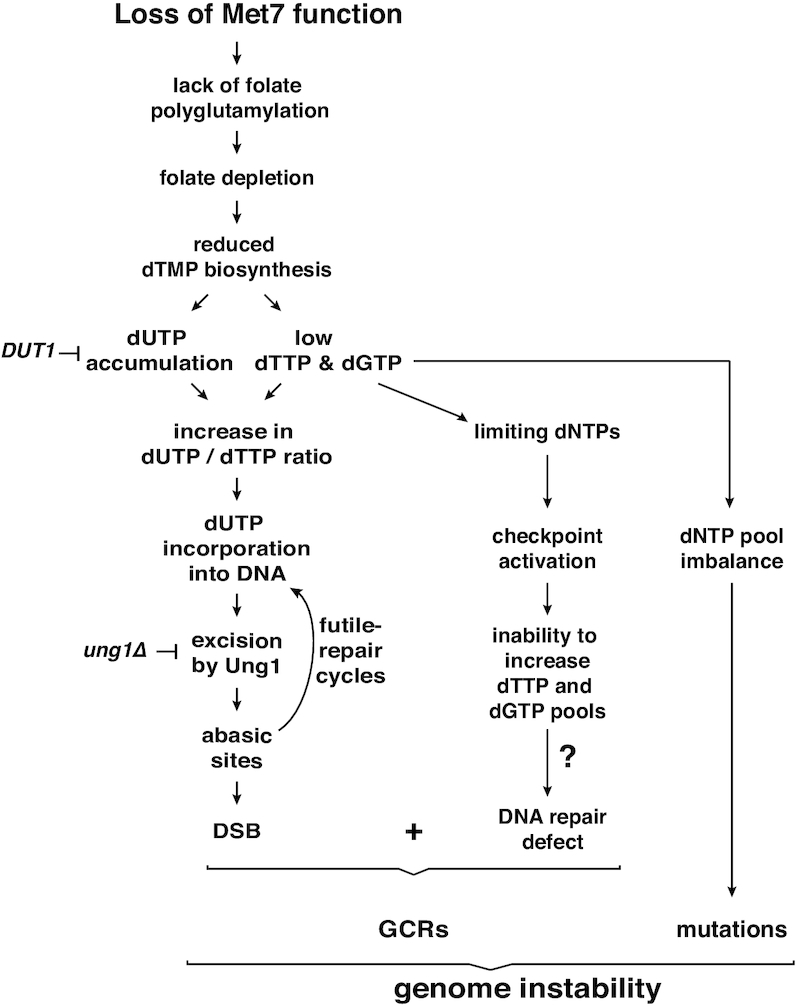
Met7 contributes to the stability of the genome. Inactivation of Met7 leads to folate depletion causing limiting dTTP and dGTP and accumulation of dUTP. A high dUTP/dTTP ratio promotes uracil incorporation into DNA. Uracil is removed from DNA by Ung1, however the high dUTP/dTTP ratio leads to futile uracil excision/incorporation cycles resulting in abasic sites and DSBs. Limiting dNTPs (dTTP and dGTP) may also affect DNA repair capacity and increase the frequency of mutations.

This study sheds light on the understanding of *met7Δ*’s pleiotropic phenotype. Based on our results, we propose that dNTP limitations observed in the *met7Δ* mutant give rise to the S-phase delay, the DNA damage checkpoint activation and potentially also for the short telomere phenotype previously reported for this mutant (34–36). Our results are in agreement with a model in which the absence of Met7 causes reduced folate pools, preventing the conversion of dUMP into dTMP and consequently the synthesis of dTTP (Figure 5). The severe genome instability phenotype in the *met7Δ* mutant occurs presumably as a combinatorial effect of imbalanced dNTP pools, that may interfere with DNA repair transactions, and increased accumulation of dUTP, resulting in a high dUTP/dTTP ratio that will cause frequent uracil incorporation into DNA, compromising both mitochondrial and nuclear DNA integrity.

## Supplementary Material

gkz1006_Supplemental_File
