# Supplementary material for: Inactivation of folylpolyglutamate synthetase Met7 results in genome instability driven by an increased dUTP/dTTP ratio
Source: Nucleic Acids Res. 2019 Oct 24;48(1):264–77. doi: 10.1093/nar/gkz1006 (PMC7145683; doi:10.1093/nar/gkz1006)
Supplement: gkz1006_Supplemental_File [file gkz1006_supplemental_file.pdf]

# Supplementary data

# Inactivation of folylpolyglutamate synthetase Met7 results in genome instability driven by an increased dUTP/dTTP ratio

Tobias T. Schmidt[1,2], Sushma Sharma[3], Gloria X. Reyes[1], Anna Kolodziejczak[1,2], Tina Wagner[4], Brian Luke[4,5], Anders Hofer[3], Andrei Chabes[3,6] and Hans Hombauer[1,*]

[1]DNA Repair Mechanisms and Cancer, German Cancer Research Center (DKFZ), Heidelberg, D-69120, Germany, [2]Faculty of Bioscience, Heidelberg University, Heidelberg, D-69120, Germany, [3]Department of Medical Biochemistry and Biophysics, Umeå University, Umeå, SE-901 87 Sweden, [4]Institute of Developmental Biology and Neurobiology, Johannes Gutenberg Universität, 55128 Mainz, Germany, [5]Institute of Molecular Biology (IMB), 55128 Mainz, Germany, [6]Laboratory for Molecular Infection Medicine Sweden (MIMS), Umeå University, SE-901 87, Umeå, Sweden.

* To whom correspondence should be addressed. Tel: +49 6221 42 3239; Fax: +49 6221 42 3237;
Email: h.hombauer@dkfz.de

## Table of contents

**Supplementary Experimental Procedures**

**Supplementary Figure S1.** Pathways of the *de novo* dNTP biosynthesis in *S. cerevisiae*

**Supplementary Figure S2.** Quantification of dUTP levels in yeast extracts.

**Supplementary Figure S3.** Met7 expression is constant throughout the cell cycle and it is not affected by DNA damage or replication stress.

**Supplementary Figure S4.** Characterization of *dut1-1* mutant strains.

**Supplementary Figure S5.** Growth rates analysis of WT and *dut1-1* mutants.

**Supplementary Table S1.** List of single gene deletions resulting in increased mutator phenotypes.

**Supplementary Table S2.** NTP and dNTP concentrations in cell extracts of the indicated yeast strains measured by SAX-HPLC and RP-HPLC.

**Supplementary Table S3.** NTP and dNTP concentrations in several mutant strains.

**Supplementary Table S4.** *CAN1* mutation spectrum of WT and *met7Δ*.

**Supplementary Table S5.** *S. cerevisiae* strains used in this study.

**Supplementary Experimental Procedures**

**Media, strains and plasmids.** *S. cerevisiae* strains were grown at 30°C in yeast extract-peptone-dextrose (YPD) medium (1% Bacto yeast extract (Becton Dickinson), 2% Bacto peptone (Becton Dickinson) and 2% glucose) or in synthetic dropout (SD) medium (0.67% Difco yeast nitrogen base without amino acids (Becton Dickinson), 2% glucose and supplemented with the appropriate amino acid dropout mix). To identify mutations that may cause a *petite* phenotype, cells were grown in yeast extract-peptone-glycerol (YPG) medium (1% Bacto yeast extract (Becton Dickinson), 2% Bacto peptone (Becton Dickinson) and 3% glycerol). For the *CAN1* inactivation assay, plates were prepared in SD medium lacking arginine (Arg), supplemented with 60 mg/L canavanine (Sigma). For the GCR assays, plates were prepared using SD medium lacking Arg and uracil (Ura) supplemented with 60 mg/L canavanine, 1 g/L 5-FOA (US Biological) and 50 mg/L Ura (Sigma). 5-FOA plates were prepared in SD medium lacking uracil supplemented with 1 g/L 5-FOA and 50 mg/L Ura. Antibiotics were used at the following final concentrations: 200 µg/mL geneticin (Santa Cruz Biotechnology), 300 µg/mL hygromycin B (Thermo Fisher Scientific) and 100 µg/mL nourseothricin (clonNAT, Werner BioAgents). Drugs HU (H8627, Sigma) and phleomycin (P9564, Sigma) were used at the indicated concentrations to induce DNA replication stress or DNA damage, respectively.

To generate strains containing the *dut1-1* allele (at the chromosomal *DUT1* locus) we used the integrative vector pHHB1094 generated as following. The *DUT1* gene, including 1 kb upstream and 752 nt downstream of *DUT1* was amplified from genomic DNA with primers 5'-CAT GAT GGA TCC CAT GCC CCA TCT CCA CGC TC-3' and 5'-CAG ACC CTA TTA GGA GCC C-3'. The introduced BamHI site in combination with an XhoI site downstream of *DUT1* was used to clone the amplified fragment into pRS306 (40) to generate pRS306-*DUT1* (pHHB1093). The *dut1-G82S* mutation (24) was introduced into pHHB1093 by site-directed mutagenesis using primers 5'-GAA AAA CGG TAT CCA AAC CGG TGC TAG TGT TGT CGA CAG AGA TTA CAC CGG-3' and 5'-CCG GTG TAA TCT CTG TCG ACA ACA CTA GCA CCG GTT TGG ATA CCG TTT TTC-3' to generate pRS306-*dut1-1* (pHHB1094). Next, the pHHB1094 plasmid was linearized with HindIII (New England Biolabs) and transformed into RDKY3686 for integration at the chromosomal *DUT1* locus, selected in Ura- medium and then counter-selected on 5-FOA containing plates (pop-in/pop-out strategy) generating HHY6650. The presence of the *dut1-G82S* mutation, as well as the absence of unwanted mutations, was confirmed by sequencing. Next, the HHY6650 was crossed against HHY6451 to generate HHY6646.

**Yeast cell lysates and immunoblotting.**

*S. cerevisiae* whole-cell protein extracts were generated as previously described (38) and were analyzed on 7%, 8% or 10% SDS-PAGE, or 4-15% Mini-PROTEAN TGX precast gels (Bio-Rad) followed by immunoblotting. The following antibodies were used: rabbit polyclonal anti-Clb2 (1:1,000, sc-9071, Santa Cruz Biotechnology), mouse monoclonal anti-c-Myc (1:1,000, 9E10, Millipore), mouse monoclonal anti-Pgk1 (1:20,000, 22C5D8, Invitrogen), mouse monoclonal anti-Rad53 (1:1,000, EL7.E1, Abcam), rabbit polyclonal anti-Rnr1 (1:40,000, AS09576, Agrisera), rabbit polyclonal anti-Rnr2 (1:30,000, AS09575, Agrisera), rabbit polyclonal anti-Rnr3 (1:1,000, AS09574, Agrisera) and guinea pig polyclonal anti-Sic1 (1:10,000, this study, details below). The rat

monoclonal YL1/2 antibody (YL1/2, 1:40,000, Sigma) was used to probe against tubulin and Rnr4. Pgk1 and tubulin were used as loading controls. Horseradish peroxidase (HRP)-linked to rabbit anti-guinea pig IgG (A60-211P, Bethyl Laboratories), HRP-linked sheep to anti-mouse IgG (NA9310, GE Healthcare), HRP-linked donkey to anti-rabbit IgG (NA934, GE Healthcare) and HRP-linked to goat anti-rat IgG (401416, Calbiochem) were used as secondary antibodies at a dilution of 1:10,000. Western blots were developed using Immobilon Western Chemiluminscent HRP substrate (Millipore) and imaged using Super RX-N Fuji medical x-ray films (Fujifilm) and an Optimax TR X-ray film processor (Protec) or using Fusion Solo S (Vilber).

**Generation of Sic1 antibodies.** To raise antibodies against full-length yeast Sic1 protein we expressed 6xHis-Sic1 from pET28c-Sic1 (kind gift from Dr. Gislene Pereira) in *E. coli BL21 (DE3)* (Cat#200131, Agilent Technologies) followed by Ni-NTA affinity purification (30210, Qiagen). Two guinea pigs were immunized with purified 6XHis-Sic1 at the Antibody Unit of the DKFZ Genomics and Proteomics Core Facility. The specificity of serum was tested by immunoblotting.

**α-factor arrest and release.**

For α-factor arrest and release experiments, HHY6644 was grown overnight in YPD medium. The logarithmically growing control cultures were prepared as described earlier (38). To arrest cells in G1 with α-factor, 2 mL of the overnight culture was washed with sterile water twice and cells were incubated in 20 mL YPD medium containing 0.1 µg/mL α-factor (RP01002, GenScript) at 30 °C for 3 hours with shaking. Next, cells were washed with sterile water twice, released in 20 mL YPD medium containing 15 µg/mL nocodazole (T2802, TargetMol) and grown at 30 °C with shaking. Samples for DNA content analysis and cell lysates were taken every 10 min. Cell cycle progression was analyzed by DNA content using FACS and by SDS-PAGE/immunoblotting.

**Live-cell imaging of Ddc2-GFP foci.**

Exponentially growing cells were washed, resuspended and placed on agar pads, covered with a coverslip and sealed with valap (a 1:1:1 mixture of Vaseline, lanolin and paraffin by weight). Cells were imaged on a Leica SP5 confocal microscope, using an Argon laser, a 63x1.4NA objective, and a high resonance scanner @8kHz frequency. Ten 0.4 µm z sections were acquired and image processing, such as maximum intensity projections, were performed using ImageJ. The data represent the percentage of cells with foci. Three independent biological replicates were analyzed per genotype. Data is presented in box-plots with whiskers (showing the $25^{th}$ and $75^{th}$ percentile) and dots represent outliers. The line inside the box represents the median of cells with foci. Mann-Whitney test was used to compare Ddc2-GFP foci abundance in WT and *met7Δ*.

***CAN1* mutation spectra analysis.** The *CAN1* gene of independent $Can^R$ colonies was amplified from genomic DNA by PCR and sequenced (GATC) as described in (26). Sequences were analyzed with Lasergene 12 (DNASTAR). Mutation spectra and mutational hotspots were compared by Fisher's exact test in R. Values of $p \leq 0.05$ were defined as significantly different. Full *CAN1* mutation spectra for the reported strains are available upon request.

**Phleomycin sensitivity and *petite* phenotype.** To test for phleomycin sensitivity yeast cultures were grown overnight in YPD medium. Next day, cultures were 10-fold serially diluted and spotted on YPD agar plates either containing or not 2.5µg/ml phleomycin. To assess for a *petite* phenotype, strains were grown and serially diluted as before, but were spotted on YPD and YPG agar plates for comparison. Spotted plates were incubated at 30 °C for 4-5 days and imaged using the GelDoc system (Bio-Rad).

**Supplementary Figure S1**

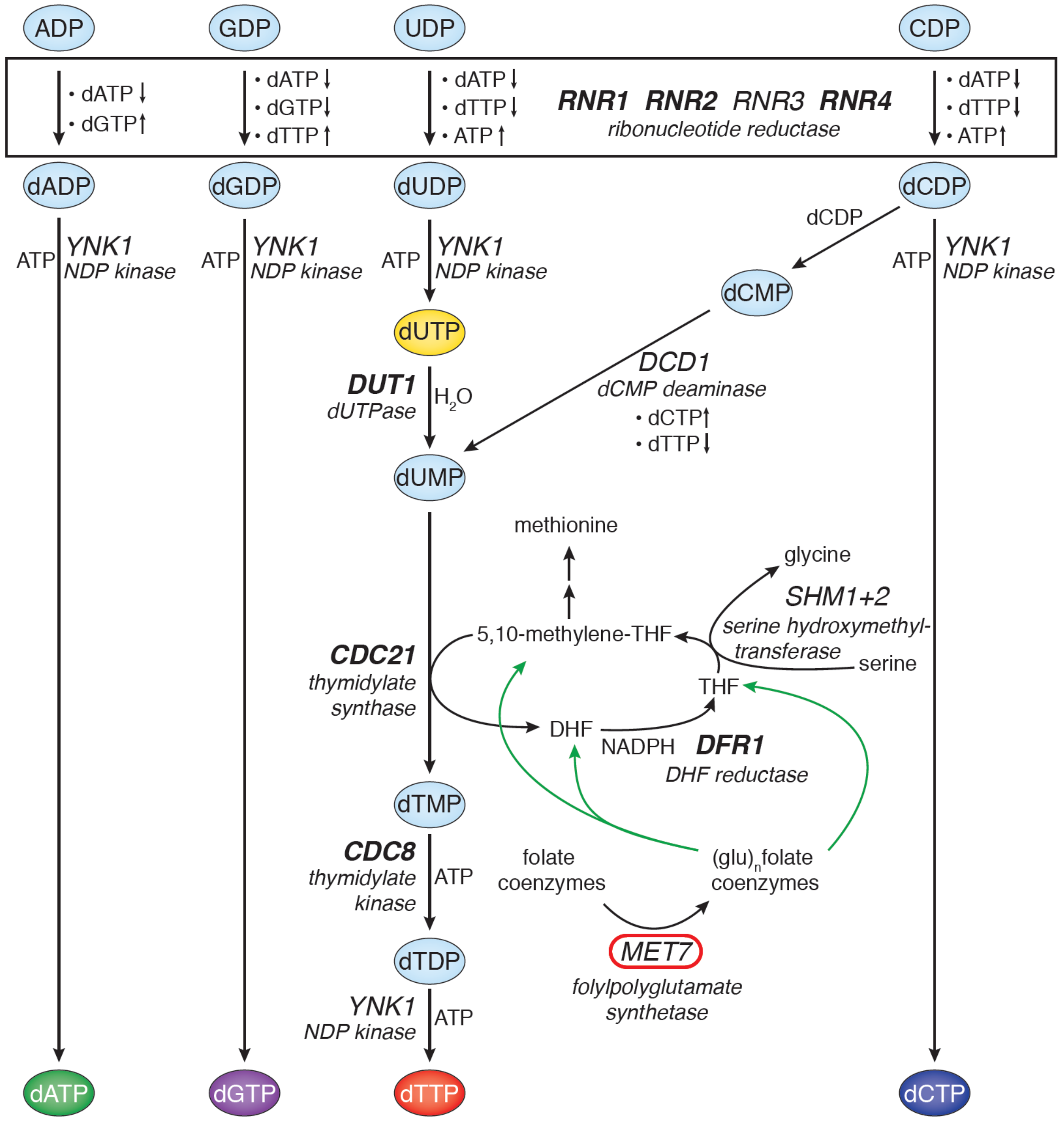


Supplementary Figure S1. Pathways of the *de novo* dNTP biosynthesis in *S. cerevisiae* (adapted from (26, 85)). Essential genes are shown in bold (*RNR1, RNR2,* and *RNR4* are non-essential genes in certain yeast genetic backgrounds). Met7 (encircled in red) catalyzes the polyglutamylation of folate coenzymes (indicated by green arrows).

## Supplementary Figure S2

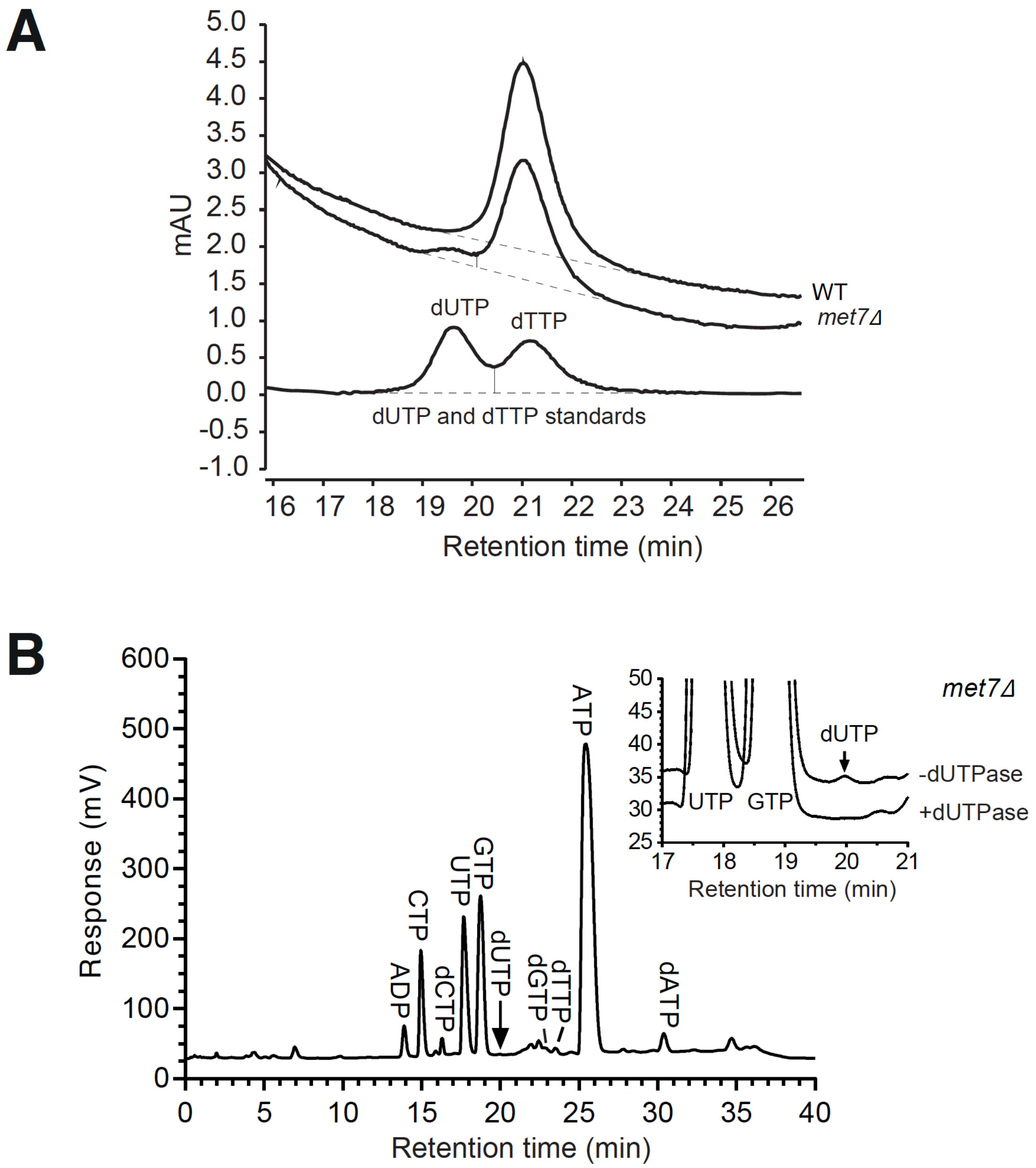


Supplementary Figure S2. Quantification of dUTP levels in yeast extracts. (A) HPLC-SAX chromatography showing that dUTP is only partially separated from dTTP. As shown in the figure, dUTP was measurable in the *met7Δ* mutant but was masked by dTTP at lower levels (WT). (B) RP chromatography procedure used for the quantification of NTPs, ADP, dCTP and dUTP. The figure shows a chromatogram from the *met7Δ* extract. The dUTP peak is magnified in the inset with a control sample treated with recombinant dUTPase shown below. The two chromatograms in the inset are plotted 5 mV apart to visualize the disappearance of the dUTP peak. Due to small interfering peaks from co-purifying metabolites, the dGTP, dTTP and dATP pools were not quantified by the RP method.

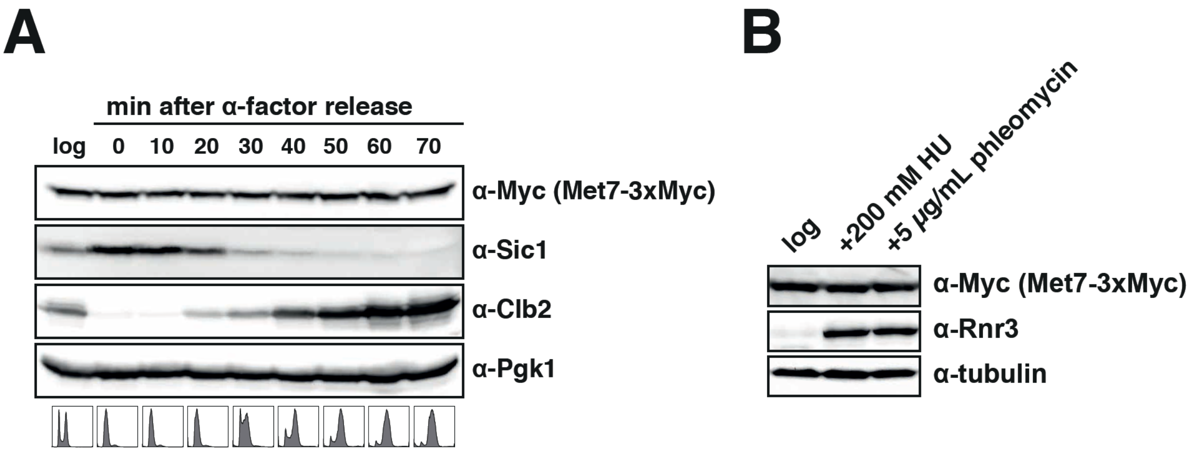


Supplementary Figure S3. Met7 expression is constant throughout the cell cycle and it is not affected by DNA damage or replication stress. (A) Cells expressing Met7-3Myc (C-terminal tagged at the endogenous locus) were arrested in G1 phase with α-factor and released into YPD containing 15 µg/mL nocodazole. Samples were collected at 10 minutes intervals for DNA content analysis and whole cell lysates. Lysates were analyzed by SDS-PAGE and immunoblotting with antibodies against Myc (for Met7-3xMyc), Sic1 (used as G1 phase marker), Clb2 (as G2 phase marker) and Pgk1 (loading control). (B) Met7 expression is not induced upon DNA replication stress or DNA damage. Cells expressing Met7-3Myc were either grown in YPD (log) or in YPD containing either 200 mM HU or 5 µg/mL phleomycin for 3 hours. Whole cell lysates were analyzed by SDS-PAGE and immunoblotting with antibodies anti-Myc (for detection of Met7-3xMyc), anti-Rnr3 (as DNA damage-inducible control) and anti-tubulin (loading control).

## Supplementary Figure S4

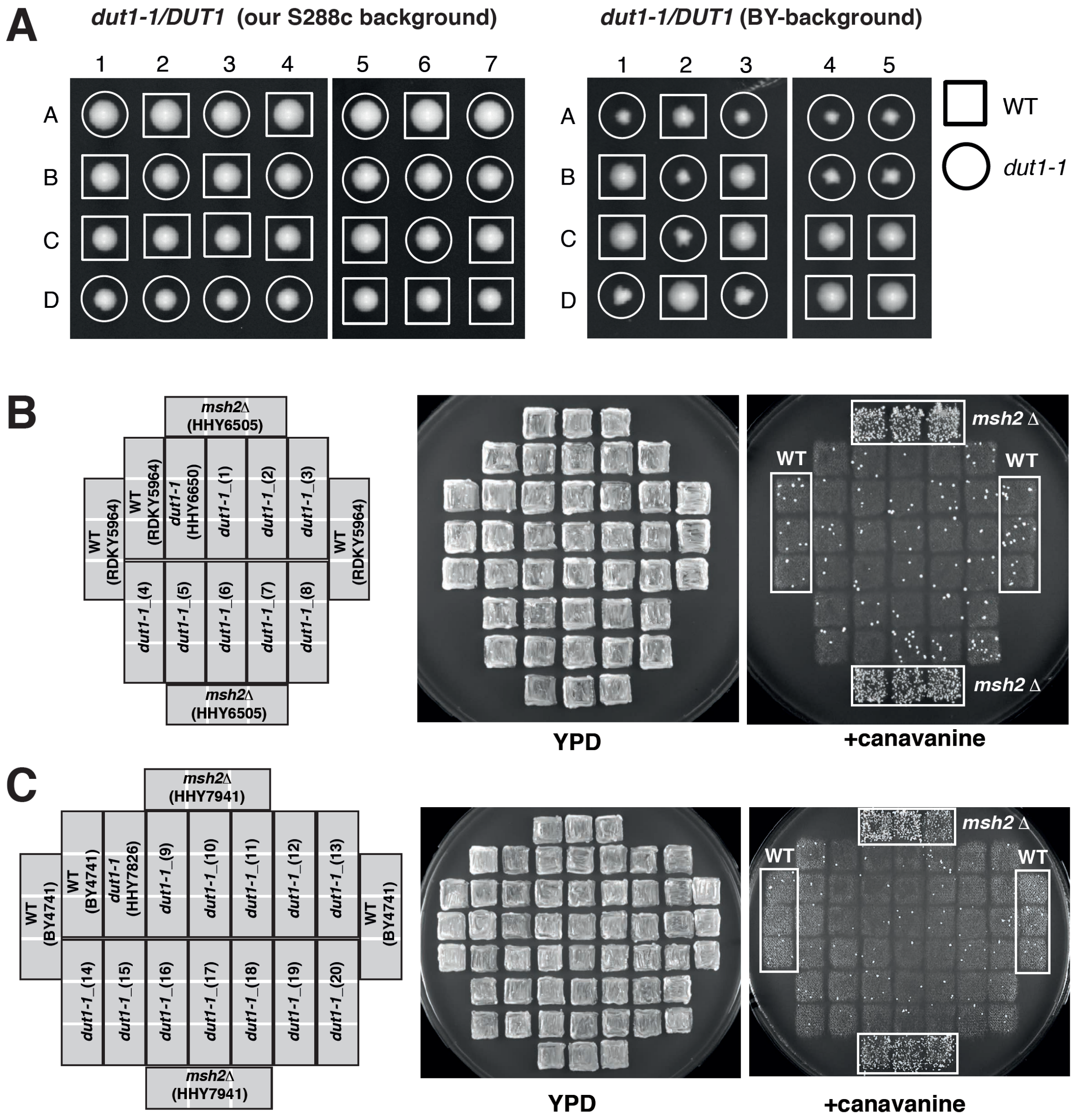


Supplementary Figure S4. Characterization of *dut1-1* mutant strains. (A) Tetrad dissection analysis of *dut1-1/DUT1* diploid strain (obtained after introducing the *dut1-1* mutation into the WT-diploid strain HHY5311 (which derives from RDKY3686/5964) (left panel) or after backcrossing a *dut1-1* x WT in the BY-4741/4742 background (right panel). Tetrads were dissected on YPD plates and grown at 30 °C for 4 days. Spores containing the *dut1-1* mutation were identified by sequencing. (B) and (C) Qualitative mutator analysis (*CAN1* gene inactivation) of *dut1-1* mutants and few additional WT and parental controls. *dut1-1* mutants (clones 1-8) obtained after tetrad dissection in HHY5311 are shown in (B), and *dut1-1* clones (9-20) in the BY-background are shown in (C). Three independent colonies for each strain were grown as patches on YPD and then replica-plated on canavanine-containing plates. Plates were incubated at 30 °C for 4-5 days. The increased abundance of canavanine-resistant colonies (relative to the WT) is indicative of a mutator phenotype. Mismatch repair deficient strains (*msh2Δ*), in their respective genetic backgrounds, were used as reference (mutator controls).

**Supplementary Figure S5**

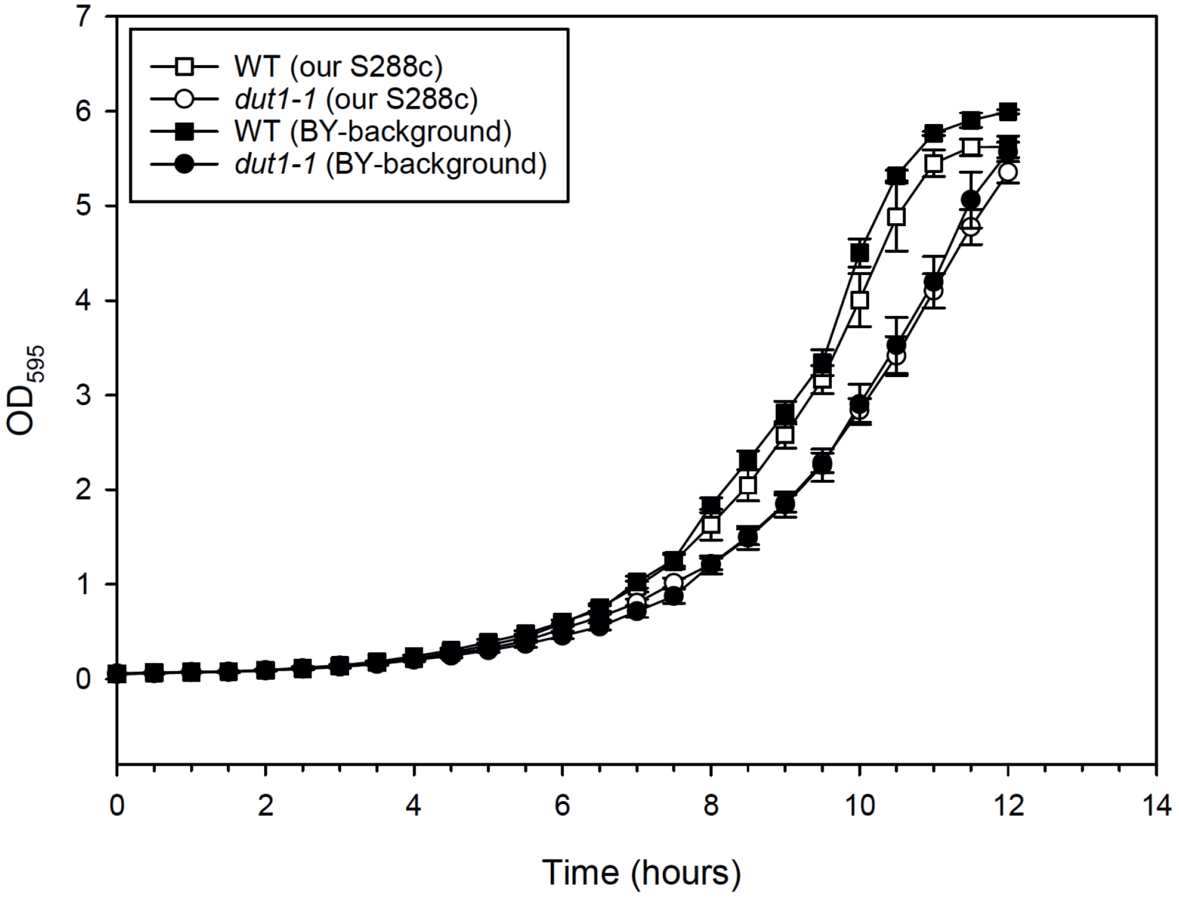


Supplementary Figure S5. Growth rates analysis of WT and *dut1-1* mutants. Yeast cultures of WT and *dut1-1* mutant strains (in our S288c or in the BY-background) were diluted to an optical density (OD) $OD_{595}$=0.05 and grown at 30 °C in YPD for 12 hours. $OD_{595}$ measurements were taken every 30min. Doubling times mentioned in the main text were calculated based on the exponential phase of each growth curve.

**Supplementary Table S1.** List of single gene deletions resulting in increased mutator phenotypes.

| Gene | *lys2-10A* | *CAN1* | Biological function | Reference |
|---|---|---|---|---|
| *CCS1* | | X | Copper chaperone, oxidative stress response | 27, 28 |
| *CSM2* | | X | Component of Shu complex, error-free DNA repair | 27, 28 |
| *ELG1* | X | X | Subunit of RFC1-like complex, DNA replication and genome integrity | 27, 28 |
| *EXO1* | X | X | 5'-3' exonuclease and flap endonuclease, DSB repair, error-free PRR and MMR | 56 |
| *MET7* | | X | Folylpolyglutamate synthetase | This study |
| *MLH1* | X | X | MMR | 27, 28 |
| *MLH3* | X | | MMR, meiotic recombination | 57 |
| *MMS2* | | X | Ubiquitin-conjugating enzyme, error-free PRR | 27, 28 |
| *MPH1* | | X | DNA helicase | 28 |
| *MRE11* | | X | Nuclease subunit of MRX complex in DSB | 27, 28 |
| *MSH2* | X | X | MMR | 27, 28 |
| *MSH3* | X | X | MMR | 27, 28 |
| *MSH6* | X | X | MMR | 27, 28 |
| *OGG1* | | X | 8-oxoguanine DNA glycosylase, BER | 27, 28 |
| *PIF1* | | X | DNA helicase | 27, 28 |
| *PMS1* | X | X | MMR | 27, 28 |
| *PSY3* | | X | Component of Shu complex, error-free DNA repair | 27 |
| *RAD1* | | X | Single-stranded DNA endonuclease, NER, DSB | 28 |
| *RAD4* | | X | NER | 55 |
| *RAD5* | | X | DNA helicase, PRR | 27, 28 |
| *RAD10* | | X | Single-stranded DNA endonuclease, NER, DSB | 58 |
| *RAD14* | | X | NER | 59 |
| *RAD17* | | X | DNA damage checkpoint | 60 |
| *RAD18* | | X | E3 ubiquitin ligase, PRR | 27, 28 |
| *RAD27* | | X | 5' to 3' exonuclease, 5' flap endonuclease, DNA replication and repair | 27, 28 |
| *RAD50* | | X | Subunit of MRX complex, DSB repair | 27 |
| *RAD51* | | X | DSB repair | 27 |
| *RAD52* | | X | DSB repair | 27, 28 |
| *RAD54* | | X | DSB repair | 27 |
| *RAD55* | | X | DSB repair | 27, 28 |
| *RAD57* | | X | DSB repair | 27, 28 |
| *RNH203* | | X | Ribonucleotide H2 subunit, ribonucleotide excision repair | 27 |
| *SHU1* | | X | Component of Shu complex, error-free DNA repair | 27, 28 |
| *SHU2* | | X | Component of Shu complex, error-free DNA repair | 27 |
| *TSA1* | | X | Thioredoxin peroxidase, oxidative stress response | 27, 28 |
| *UBC13* | | X | E2 ubiquitin-conjugating enzyme, error-free PRR | 61 |
| *UNG1* | | X | Uracil-DNA glycosylase, BER | 27, 28 |
| *XRS2* | | X | Subunit of MRX complex, DSB repair | 27 |
| *YAP1* | | X | Transcription factor, oxidative stress response | 27, 28 |

The following mutants including: *sli15Δ*, *ygr050cΔ*, *yhl005cΔ*, *yml083c, ymr166cΔ*, and *zwf1Δ* were identified as false positives. Abbreviations: base excision repair (BER), double-strand break (DSB), mismatch repair (MMR), nucleotide excision repair (NER), post-replicative repair (PRR).

**Supplementary Table S2.** NTP and dNTP concentrations in cell extracts of the indicated yeast strains measured by SAX-HPLC and RP-HPLC.

## A

| Relevant genotype | CTP | UTP | ATP | GTP |
|---|---|---|---|---|
| WT | 2374 ± 16 (1.0) | 5605 ± 32 (1.0) | 11339 ± 48 (1.0) | 3987 ± 67 (1.0) |
| *met7Δ* | 2206 ± 5 (0.9) | 4109 ± 60 (0.7) | 13697 ± 153 (1.2) | 4514 ± 3 (1.1) |
| *met7Δ pGPD-DUT1* | 2242 ± 9 (0.9) | 3915 ± 13 (0.7) | 13676 ± 57 (1.2) | 4601 ± 15 (1.2) |

| Relevant genotype | dCTP | dTTP | dATP | dGTP | dUTP |
|---|---|---|---|---|---|
| WT | 114 ± 4 (1.0) | 203 ± 6 (1.0) | 125 ± 4 (1.0) | 82 ± 4 (1.0) | nd |
| WT + hDut1 | 104 ± 1 (0.9) | 203 ± 7 (1.0) | 123 ± 4 (1.0) | 81 ± 1 (1.0) | nd |
| *met7Δ* | 302 ± 7 (2.7) | 125 ± 5 (0.6) | 497 ± 1 (4.0) | 49 ± 4 (0.6) | 9 ± 2 |
| *met7Δ* + hDut1 | 282 ± 3 (2.5) | 125 ± 6 (0.6) | 490 ± 9 (3.9) | 49 ± 6 (0.6) | nd |
| *met7Δ pGPD-DUT1* | 314 ± 1 (2.8) | 95 ± 2 (0.5) | 463 ± 1 (3.7) | 38 ± 2 (0.5) | nd |
| *met7Δ pGPD-DUT1* + hDut1 | 284 ± 1 (2.5) | 95 ± 1 (0.5) | 444 ± 5 (3.6) | 40 ± 2 (0.5) | nd |

## B

| Relevant genotype | CTP | UTP | ATP | GTP |
|---|---|---|---|---|
| WT | 2425 ± 216 (1.0) | 5839 ± 312 (1.0) | 11978 ± 928 (1.0) | 3654 ± 356 (1.0) |
| *met7Δ* | 2160 ± 218 (0.9) | 3846 ± 255 (0.7) | 13664 ± 1468 (1.2) | 4152 ± 626 (1.2) |
| *met7Δ* + hDut1 | 2122 ± 396 | 3753 ± 568 | 13305 ± 2609 | 4126 ± 1009 |

| Relevant genotype | dCTP | dUTP | ADP | ATP/ADP |
|---|---|---|---|---|
| WT | 102 ± 15 (1.0) | ≤ 1 | 692 ± 11 (1.0) | 17 |
| *met7Δ* | 306 ± 44 (3.0) | 11 ± 2 | 566 ± 45 (0.8) | 24 |
| *met7Δ* + hDut1 | 298 ± 49 (2.9) | ≤ 1 | 752 ± 4 (1.1) | 18 |

NTP and dNTP concentrations determined by SAX-HPLC (A) or RP-HPLC (B). Concentrations are indicated as pmol/$10^8$ cells and correspond to the average of two biological replicates ± standard deviation with the fold increase over WT in brackets. Extracts treated with recombinant hDut1 (+ hDut1 samples) prior the measurement are indicated. The dUTP peak was partially overlapping with dTTP with the SAX method (shown in A). "nd" means "not detectable" or below our detection limit (≤3 pmol dUTP). The dUTP pools were instead determined more accurately with the RP-HPLC method. According to this method, extracts of *met7Δ* strains contained about 10 times more dUTP than WT extracts. The RP-HPLC method also allowed to measure ADP, and the high ATP/ADP ratio in the samples indicate that there is no disturbance of the general energy metabolism in the cells. Measurements of dGTP, and to some extent dTTP and dATP, were disturbed by interfering peaks in the RP method and because of that not included in the data set.

**Supplementary Table S3.** NTP and dNTP concentrations in several mutant strains.

## A

| Relevant genotype | CTP | UTP | ATP | GTP |
|---|---|---|---|---|
| WT | 2256 ± 63 (1.0) | 5092 ± 79 (1.0) | 11151 ± 219 (1.0) | 3584 ± 80 (1.0) |
| *met7Δ* | 2137 ± 7 (0.9) | 3563 ± 27 (0.7) | 13356 ± 199 (1.2) | 4107 ± 71 (1.1) |
| *dut1-1* | 2215 ± 28 (1.0) | 5213 ± 339 (1.0) | 11247 ± 335 (1.0) | 3532 ± 82 (1.0) |
| *dcd1Δ* | 2009 ± 12 (0.9) | 5688 ± 109 (1.1) | 11392 ± 179 (1.0) | 3476 ± 36 (1.0) |
| *dut1-1 dcd1Δ* | 2269 ± 48 (1.0) | 4870 ± 65 (1.0) | 11820 ± 152 (1.1) | 3501 ± 19 (1.0) |

## B

| Relevant genotype | dCTP | dTTP | dATP | dGTP |
|---|---|---|---|---|
| WT | 79 ± 9 (1.0) | 178 ± 22 (1.0) | 107 ± 9 (1.0) | 69 ± 5 (1.0) |
| *met7Δ* | 278 ± 2 (3.5) | 110 ± 5 (0.6) | 472 ± 31 (4.4) | 36 ± 3 (0.5) |
| *dut1-1* | 114 ± 3 (1.4) | 262 ± 2 (1.5) | 161± 7 (1.5) | 88 ± 1 (1.3) |
| *dcd1Δ* | 1908 ± 90 (24.2) | 90 ± 3 (0.5) | 124 ± 3 (1.2) | 48 ± 2 (0.7) |
| *dut1-1 dcd1Δ* | 1543 ± 0 (19.6) | 165 ± 2 (0.9) | 191 ± 4 (1.8) | 55 ± 1 (0.8) |

NTP (A) and dNTP (B) concentrations (pmol per $10^8$ cells) determined by SAX-HPLC correspond to the average of two biological replicates ± standard deviation with the fold increase over WT in parentheses.

**Supplementary Table S4.** *CAN1* mutation spectrum of WT and *met7Δ*.

| | insertions / deletions | | | base substitutions | | complex* |
|---|---|---|---|---|---|---|
| **genotype** | | **mutation** | **occurrence** | **mutation** | **occurrence** | **occurrence** |
| **WT**[†] | ΔA | A6 → A5 | 1 (1) | A-T → G-C | 6 (7) | 8 (9) |
| | ΔT | T6 → T5 | 2 (2) | G-C → A-T | 18 (20) | |
| | | T2 → T1 | 2 (2) | G-C → T-A | 29 (32) | |
| | ΔC | C2 → C1 | 1 (1) | A-T → C-G | 3 (3) | |
| | | C1 → C0 | 2 (2) | A-T → T-A | 7 (8) | |
| | +T | T6 → T7 | 3 (3) | C-G → G-C | 6 (7) | |
| | | T2 → T3 | 3 (3) | | | |
| | +G | G2 → G3 | 1 (1) | | | |
| | | | **15 (16)** | | **69 (75)** | **8 (9)** |
| ***met7Δ*** | ΔA | A3 → A2 | 1 (1) | A-T → G-C | 5 (3) | 19 (10) |
| | ΔT | T6 → T5 | 4 (2) | G-C → A-T | 54 (29) | |
| | | T4 → T3 | 4 (2) | G-C → T-A | 40 (22) | |
| | | T3 → T2 | 2 (1) | A-T → C-G | 11 (6) | |
| | | T1 → T0 | 3 (2) | A-T → T-A | 14 (8) | |
| | ΔG | G4 → G3 | 2 (1) | C-G → G-C | 19 (10) | |
| | | G2 → G1 | 2 (1) | | | |
| | | G1 → G0 | 1 (1) | | | |
| | ΔC | C3 →C2 | 2 (1) | | | |
| | | C2 → C1 | 1 (1) | | | |
| | +T | T6 → T7 | 1 (1) | | | |
| | | | **23 (12)** | | **143 (77)** | **19 (10)** |

Mutation spectra analysis based on DNA sequencing of the *CAN1* gene in independent $Can^R$ mutants of WT (n=91) and *met7Δ* (n=185), shown as the number of clones containing the indicated mutations, and in parenthesis as the percentage relative to the total.

* includes: multiple mutations within 10 nucleotides, insertions or deletions of more than 1 nucleotide and duplication events.

[†] In few cases (about 1-2% of the sequenced clones) two simultaneous *CAN1* mutations (more than 100 bp apart) were found. These mutations were included in the analysis and considered as independent mutational events. *CAN1* spectrum of WT strain was taken from (26).

## **Supplementary Table S5**. *S. cerevisiae* strains used in this study.

| Name | Relevant genotype [a] | Reference |
|---|---|---|
| RDKY5964 | *MATa ura3-52 leu2Δ1 trp1Δ63 hom3-10 his3Δ200 lys2-10A* | (38) |
| HHY5298 | RDKY5964 *cyh2 Q38K hom3-10.HIS3 pMFA1-klLEU2.hphNT1.lys2-10A MLH2.klURA3 POL1.natNT2* | (26) |
| HHY5311 | *MATa/α ura3-52/ura3-52 leu2Δ1/ leu2Δ1 trp1Δ63/ trp1Δ63 hom3-10/ hom3-10 his3Δ200/ his3Δ200 lys2-10A/ lys2-10A* | This study |
| HHY6370 | RDKY5964 *met7::kanMX4* | This study |
| HHY6505 | RDKY5964 *msh2::HIS3* | This study |
| HHY7735 | RDKY5964 *kanMX4.pGPD-DUT1* | This study |
| HHY6441 | RDKY5964 *kanMX4.pGPD-DUT1 met7::klTRP1* | This study |
| HHY7737 | RDKY5964 *ung1::hphNT1* | This study |
| HHY7739 | RDKY5964 *ung1::hphNT1 met7::kl-TRP1* | This study |
| HHY1602 | RDKY5964 *rev3:: kanMX4* | This study |
| HHY6636 | RDKY5964 *rev3::natNT2 met7::klTRP1* | This study |
| HHY6707 | RDKY5964 *dut1-G82S* | This study |
| HHY7733 | RDKY5964 *dcd1::kanMX4* | This study |
| HHY7564 | RDKY5964 *dut1-G82S dcd1::kanMX4* | This study |
| RDKY3686 | *MATα ura3-52 leu2Δ1 trp1Δ63 hom3-10 his3Δ200 lys2-10A* | (37) |
| HHY2883 | RDKY3686 *ddc2-GFP.hphNT1* | This study |
| HHY6479 | RDKY3686 *ddc2-GFP.hphNT1 met7::klTRP1* | This study |
| HHY6650 | RDKY3686 *dut1-G82S* | This study |
| HHY6443 | RDKY5964 *iYEL072W::hph can1::hisG yel072w::CAN1/URA3 bar1::loxP.klLEU2.loxP* | (26) |
| HHY6445 | HHY6443 *met7::kanMX4* | This study |
| HHY6638 | HHY6443 *natNT2.pGPD-DUT1* | This study |
| HHY6447 | HHY6443 *natNT2.pGPD-DUT1 met7::klTRP1* | This study |
| HHY6451 | HHY6443 *ung1::kanMX4* | This study |
| HHY6449 | HHY6443 *ung1::kanMX4 met7::klTRP1* | This study |
| HHY6640 | HHY6443 *natNT2.pGPD-DUT1 met7::klTRP1 ung1::kanMX4* | This study |
| HHY6644 | HHY6443 *met7-3xMYC::klTRP1* | This study |
| HHY6646 | HHY6443 *dut1-G82S* | This study |
| HHY6716 | HHY6443 *dcd1::natNT2* | This study |
| HHY6648 | HHY6443 *dut1-G82S ung1::kanMX4* | This study |
| HHY6713 | HHY6443 *dut1-G82S dcd1::natNT2* | This study |
| RDKY3615 | *MATa ura3-52 leu2Δ1 trp1Δ63 his3Δ200 lys2ΔBgl hom3-10 ade2Δ1 ade8 yel069c::URA3* | (39) |
| HHY6477 | RDKY3615 *met7::HIS3* | This study |
| BY4741 | *MATa his3Δ1 leu2Δ0 met15Δ0 ura3Δ0* | Euroscarf |
| BY4742 | *MATα his3Δ1 leu2Δ0 lys2Δ0 ura3Δ0* | Euroscarf |
| HHY7826 | BY4741 *dut1-1* | This study |
| HHY7941 | BY4742 *msh2::kanMX4* | Euroscarf |

[a] All strains derived from S288c. The genotype corresponds to the listed strain with the indicated modifications.
